# Extreme metal adapted, knockout and knockdown strains reveal a coordinated gene expression among different *Tetrahymena thermophila* metallothionein isoforms

**DOI:** 10.1371/journal.pone.0189076

**Published:** 2017-12-05

**Authors:** Patricia de Francisco, Ana Martín-González, Aaron P. Turkewitz, Juan Carlos Gutiérrez

**Affiliations:** 1 Departamento de Microbiología-III, Facultad de Biología, Universidad Complutense de Madrid (UCM), Madrid, Spain; 2 Department of Molecular Genetics and Cell Biology, Cummings Life Sciences Center, University of Chicago, Chicago, Illinois, United States of America; University of Naples Federico II, ITALY

## Abstract

Metallothioneins (MT) constitute a superfamily of small cytosolic proteins that are able to bind metal cations through numerous cysteine (Cys) residues. Like other organisms the ciliate *Tetrahymena thermophila* presents several MT isoforms, which have been classified into two subfamilies (Cd- and Cu-metallothioneins). The main aim of this study was to examine the specific functions and transcriptional regulation of the five MT isoforms present in *T*. *thermophila*, by using several strains of this ciliate. After a laboratory evolution experiment over more than two years, three different *T*. *thermophila* strains adapted to extreme metal stress (Cd^2+^, Cu^2+^ or Pb^2+^) were obtained. In addition, three knockout and/or knockdown strains for different metallothionein (MT) genes were generated. These strains were then analyzed for expression of the individual MT isoforms. Our results provide a strong basis for assigning differential roles to the set of MT isoforms. *MTT1* appears to have a key role in adaptation to Cd. In contrast, *MTT2/4* are crucial for Cu-adaptation and *MTT5* appears to be important for Pb-adaptation and might be considered as an “alarm” MT gene for responding to metal stress. Moreover, results indicate that likely a coordinated transcriptional regulation exists between the MT genes, particularly among *MTT1*, *MTT5* and *MTT2/4*. *MTT5* appears to be an essential gene, a first such report in any organism of an essential MT gene.

## Introduction

Metals are natural components of the Earth's crust, and some are essential in low concentrations for cellular metabolism and growth. Examples include metal co-factors in enzymatic reactions (e.g., metalloenzymes), and stabilization of some biological molecules. On the other hand, many non-essential metals are known as the most abundant, persistent and toxic inorganic pollutants on our planet [1]. Metal toxicity can arise from interaction with biomolecules, such as proteins or nucleic acids, whose native structure and function may be thereby altered. Moreover, metal toxicity often produces oxidative stress that generates reactive oxygen species, which can perturb protein structures or enzymatic functions, impair DNA repair, and inhibit cell proliferation and differentiation processes, in some cases leading to necrotic or apoptotic cell death [2–4].

Organisms have evolved a range of mechanisms to reduce metal toxicity. One of the most universal cellular detoxification processes is the chelation of metal cations by specific oligopeptides (glutathione, phytochelatins) or proteins (metallothioneins). This mechanism allows the sequestration of metallic ions by the -SH groups of the cysteine residues and their intracellular storage in vacuoles or cytoplasmic inclusions. Then, these metal-protein complexes are expelled from the cell as non-toxic or rarely toxic compounds [5].

Metallothioneins (MTs) constitute a superfamily of small cytosolic proteins that are able to bind metal cations through numerous cysteine (Cys) residues [5–6]. These proteins have been reported in a wide variety of organisms: prokaryotic and eukaryotic microorganisms, plants, invertebrates and vertebrates. The range of specific functions of these proteins is not yet clear. Among the proposed functions are the regulation of essential-metal homeostasis [7], metal detoxification [8], protection against oxidative stress [9], and regulation of cell proliferation and apoptosis [10]. In mammals, MTs function in protection against neurodegenerative diseases [11] and in several cellular differentiation processes [12]. Broadly speaking, MTs have been considered as multifunctional proteins [10, 13] and as key elements of the cellular integrated stress response program [14]. The majority of MT gene expression studies, carried out in many different organisms, have reported that these genes are regulated primarily at the transcriptional level [10, 15], although some evidence of regulation at translational level exists [16]. That is, MT genes are often expressed constitutively but are up-regulated upon exposure to metals or other environmental stressors [17–23]. MTs have not been reported to be essential, but they are likely to provide survival advantages under some stress conditions [24, 25].

Ciliate MTs have unique features with respect to MTs from other organisms. The proteins are longer and richer in Cys residues, conferring a larger metal binding capacity compared to classic MTs [6]. The MTs in *T*. *thermophila* belong to family 7, which can be resolved into two well-characterized subfamilies. Subfamily 7a corresponds to CdMT (cadmium-metallothioneins), while 7b corresponds to CuMT (copper-metallothioneins). These two subfamilies differ mainly in their distributions of conserved cysteines, and in their preferential transcriptional induction by cadmium or copper, respectively [5, 6, 26]. *T*. *thermophila* has five MT isoforms: three CdMT (MTT1, MTT3 and MTT5) and two CuMT (MTT2 and MTT4) [20]. Previously, it was found that the individual CdMT genes have different induction patterns, suggesting functional diversification [20]. One basis for this differential transcription may be found in the promoter regions: the five *T*. *thermophila* MT genes have different numbers of a conserved MTCM1 motif, which is relevant for regulation by AP-1 transcription factors (bZIP) [6, 20]. The *MTT5* promoter has 13 MTCM1 motifs and this gene shows strongest induction after metal exposure, while the *MTT3* promoter has 2 MTCM1 motifs and is the most weakly induced *T*. *thermophila* MT gene [20]. The gene expression results [20] were corroborated by Espart and colleagues [27] who analysed the metal binding preferences of the *T*. *thermophila* MTs. MTT1 and MTT5 isoforms preferentially bind Cd^2+^ ions, while MTT3 preferentially binds Zn^2+^. Both MTT2 and MTT4 isoforms mainly form homometallic Cu-complexes [27].

Microorganisms can acquire stress tolerance and novel metabolic abilities when they are exposed to selective pressure [28]. An experimental approach to study this phenomenon has been called "evolutionary engineering" [29]. Studies in which cells are forced to adapt to increasing levels of specific stressors can provide insights into the physiological and genetic mechanisms involved in cellular responses to environmental stressors. As the stressor intensity is progressively increased, the cells’ deployment of protective mechanisms against toxicity is likely to involve enhancing a set of mechanisms involved in the normal cellular response, which can thus be more easily detected and studied through such studies. Experiments of this type have been carried out in diverse organisms to artificially evolve resistance to abiotic stressors including high salt [30], copper, cadmium [28, 31] or alcohols [32]. There are also examples of adapted microorganisms that have developed new properties with industrial applications, an example being yeasts able to ferment xylose [33] or lactose [34].

Knockout (KO) strains are basic tools for assessing the function and relevance of specific genes [35]. Some genes are designated essential, because their total knockout is incompatible with cell viability. In such cases, gene function can be studied by reducing gene copy number or expression to generate a knockdown (KD) strain, using specific interference RNAs or other methods.

Even in well-studied model organisms, there remain many unanswered questions concerning differential expression of MT isoforms and the genetic bases of adaptation to severe metal stress. The ciliate *T*. *thermophila* is a very useful model for addressing these issues in eukaryotic microorganisms [5]. The main aim of this study was to examine the specific functions and transcriptional regulation of the five MT isoforms. In particular, we used qRT-PCR (quantitative reverse transcription polymerase chain reaction) to analyze the expression patterns of the five MT genes in a set of *T*. *thermophila* strains: the reference strain SB1969, two strains engineered to over-express *MTT1* or *MTT5* gene [36], three metal-adapted strains (adapted to extreme Cd^2+^, Cu^2+^ or Pb^2+^ concentrations) and three KO and/or KD strains targeting *MTT1* and/or *MTT5*.

## Materials and methods

### Strains and culture conditions

*Tetrahymena thermophila* strain CU428 (*mpr1-1/mpr1-1*; pm-S, mp-S, mt VII) was used to obtain the KO and/or KD strains. SB1969 (*chx1-1/chx1-1*, *mpr1-1/mpr1-1*; pm-S, cy-S, mt II), kindly supplied by Dr. E. Orias (University of California, Santa Barbara, USA), was used as a control in gene expression studies, and was also used to obtain the metal-adapted strains. Micronuclear genotypes of these strains are homozygous *mpr1-1* (6-methyl-purine resistant) or *chx1-1* (cycloheximide resistant), respectively. Their macronuclear phenotypes are pm-S (paromomycin sensitive), mp-S (6-methyl-purine sensitive) or cy-S (cycloheximide sensitive) and their mating types (mt) are VII or II. Strains GFPMTT1 and GFPMTT5 harbour multi-copy plasmids bearing the constructs P_MTT1_::GFP::MTT1 or P_MTT1_::GFP::MTT5, which over-express *MTT1* or *MTT5*, respectively [36].

Cells were axenically grown in PP210 medium [2% w/v proteose peptone (Pronadisa), supplemented with 10 μM FeCl_3_ and 250 μg/ml of streptomycin sulphate (Calbiochem) and penicillin G (Sigma)] or SPPA medium [2% proteose peptone (Difco), 0.1% yeast extract (Difco), 0.2% glucose (Sigma), 0.003% Fe-EDTA (Sigma), supplemented with 250 μg/ml of streptomycin sulphate, 250 μg/ml of penicillin G (Sigma) and 0.25 μg/ml of amphotericin B (Sigma)], and maintained at a constant temperature of 30 ± 1°C. We added 12 μg/ml of paromomycin sulphate (Sigma) in the GFPMTT1 and GFPMTT5 cultures to maintain the multi-copy plasmid.

### Extreme metal-adaptation of *T*. *thermophila* strains

Three *T*. *thermophila* metal-adapted strains were generated: Cd-adap (cadmium-adapted strain), Cu-adap (copper-adapted strain) and Pb-adap (lead-adapted strain). These metal-adapted strains were obtained after exposing strain SB1969 in PP210 medium to increasing metal concentrations [Cd^2+^ (CdCl_2_, Sigma), Cu^2+^ (CuSO_4_ ∙5 H_2_O, Sigma) or Pb^2+^ (Pb(NO_3_)_2_, Sigma)]. The adaptation process consisted on increasing 10 μM the metal concentration every week, and during about 15 months of increasing metal concentrations the survivor cells were selected until achieving their maximum tolerated concentration (MTC). Then, cells were maintained to this MTC during more than two years to complete their metal adaptation. The MTC was achieved at different time periods depending on type of metal. Metal-adapted strains were permanently maintained in PP210 medium with the MTC of the relevant metal (indicated as MTC cultures).

### Isolating MT knockout / knockdown strains

**Fig 1:**
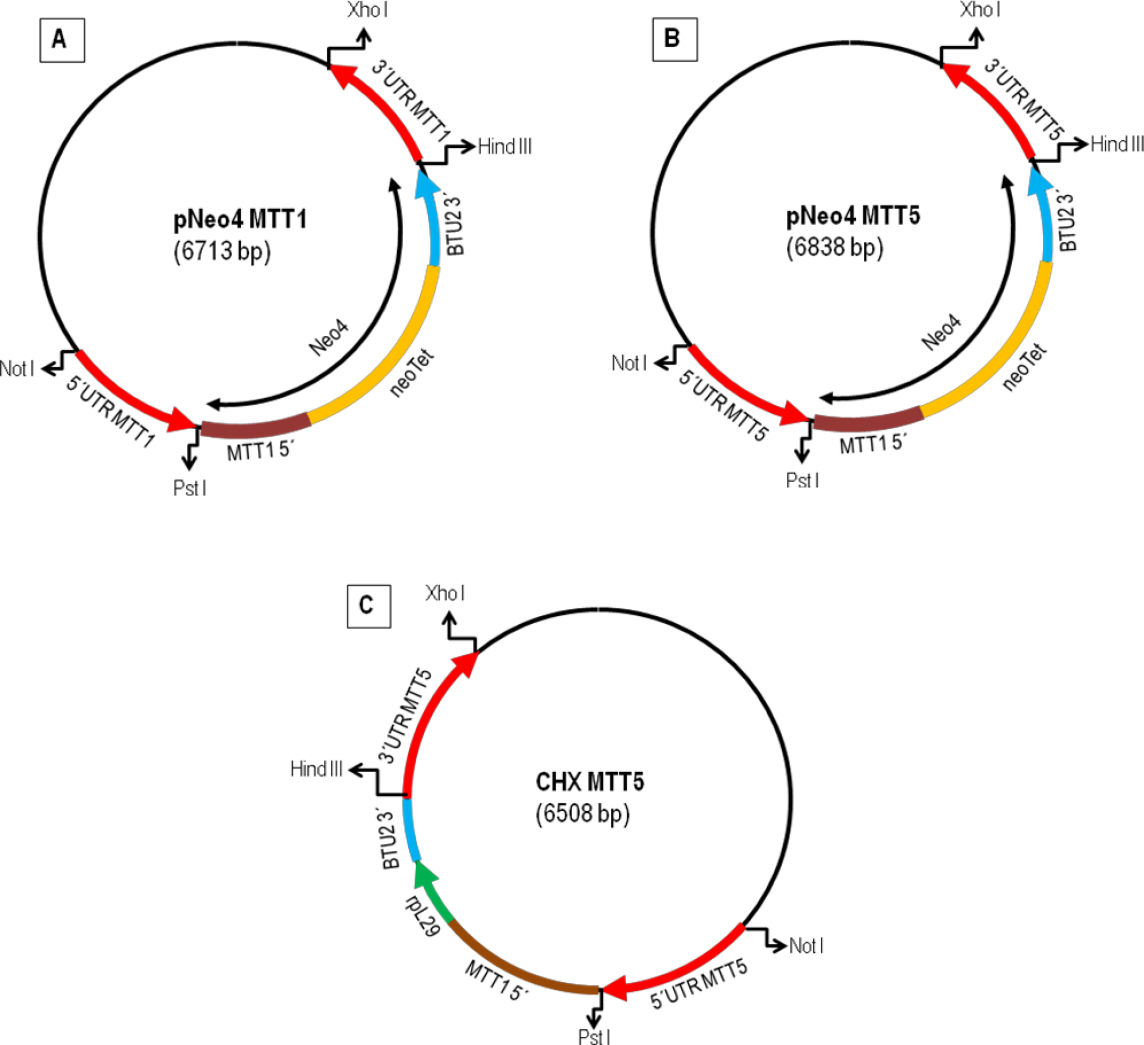
Schematic representation of the plasmid constructs used to obtain knockout and/or knockdown strains. (A): plasmid construct (pNeo4MTT1) used for MTT1KO strain. (B): plasmid construct (pNeo4MTT5) used for MTT5KD. (C): plasmid construct (pCHXMTT5) used for obtaining MTT1KO + MTT5KD. neoTet: neomycin/paromomycin resistance gene. rpl29: cycloheximide resistance gene. BTU2 3': part of the 3'UTR region of the β-tubulin 2 gene.

Constructs in Fig 1 were obtained by the directional cloning of the 5' and 3' UTRs of the *T*. *thermophila MTT1* and *MTT5* genes in the pNeo4 and rpl29 vectors. First, we amplified the 5' and 3' UTRs regions of the *MTT1* and *MTT5* genes using primers *5UTRMTT1A/1B* and *3UTRMTT1A/1B* for *MTT1* and *5UTRMTT5A/5B* and *3UTRMTT5A/5B* for *MTT5* (S1 Table, Fig 1). To obtain single *MTT1* or *MTT5* knockouts, we introduced each amplified UTR into pNeo4 after cutting with NotI HF and PstI (New England Biolabs) for the 5' UTRs and HindIII and XhoI (New England Biolabs) for the 3' UTRs, to obtain pNeo4::MTT1 and pNeo4::MTT5. These contain the neomycin/paromomycin resistance genes under the control of the cadmium-inducible *MTT1* promoter (neo4 cassette) [37] and both flanked by the UTR regions of *MTT1* or *MTT5*, respectively (Fig 1). To isolate a double KO (MTT1KO + MTT5KO) beginning with a complete MTT1KO, we prepared a *MTT5* knockout construct, pCHXMTT5, using the rpl29 vector. The UTR regions of *MTT5* were incorporated into the rpl29 vector using NotI HF and PstI for the 5' UTR and HindIII and XhoI for the 3' UTR. The rpl29 vector contains the cycloheximide resistance gene (*rpl29*) under the control of the *MTT1* promoter and both were flanked by the *MTT5* UTRs (Fig 1). Before transformation, the correct constructions were verified by sequencing.

Plasmids were introduced by biolistic transformation. Gold bombardment particles (Seashell Technology) at 30 mg/ml concentration were coated, immediately before use, with 5 μg of DNA/1 mg gold particles. CU428 (3 x 10^5^ cells/ml) were starved for 16-18h (TrisHCl buffer 0.01M pH 6.8). The starved cells (30 ml) were centrifuged at 1,100g for 1 min and resuspended in 1 ml of Tris HCl buffer. Then, 1ml cell sample was evenly spread on a sterile circular filter paper and immediately bombarded with the DNA coated gold particles at 900 psi using the DuPont Biolistic PDS-1000/He, Particle Delivery System (Biorad) [38]. Cells were then resuspended in 50 ml of 2% proteose peptone (PP2) medium and maintained with shaking at 30°C for 4-5h. Transformants were selected with 120 μg/ml of paromomycin with 1 μg/ml CdCl_2_, which drives neo4 expression from the *MTT1* promoter. For selecting double knockouts, transformant cells were selected with 12 μg/ml of cycloheximide with 1 μg/ml CdCl_2_. Cells were transferred daily during at least three weeks under increasing concentrations of paromomycin or cycloheximide, while reducing CdCl_2_ to drive phenotypic assortment. By this procedure, *MTT1* or *MTT5* genes were progressively substituted by the resistance cassette, initially integrated at a single site by homologous recombination, until all the *MTT1* or *MTT5* macronuclear gene copies were eliminated (knockout fixation).

We checked that knockout was complete by maintaining the putative KO cells without the antibiotic selective agent for one week. We then isolated total RNA, reverse-transcribed it to obtain the corresponding cDNAs, and carried out standard PCR to check if there were remaining copies of *MTT1* or *MTT5* in the macronuclear genome. The MTT1KO was complete but in the putative MTT5KO strain it was not possible to obtain cells lacking all copies of *MTT5* gene; thus we consider it as a knockdown (MTT5KD) strain. Similarly, we generated a MTT1KO + MTT5KD strain. Both knockdown strains are inherently unstable, because without selective pressure the gene copy number of MTT5 can increase. To prevent this, we maintained the cells in 800 μg/ml of paromomycin (for MTT5KD) or 60 μg/ml of cycloheximide (for MTT1KO + MTT5KD).

### Metal stress treatments

Cells were exposed for 1 or 24h to Cd^2+^, Cu^2+^ or Pb^2+^. Control, KO and KD strains were treated with 44.5 μM (Cd^2+^), 315 μM (Cu^2+^) or 965 μM (Pb^2+^), while metal-adapted, GFPMTT1 and GFPMTT5 strains were exposed to the MTC for each metal: 115 μM Cd^2+^, 4 mM Cu^2+^ or 5.5 mM Pb^2+^. In some experiments with the metal adapted strains, cells were maintained for 24h in PP210 without any added metal and then were exposed for 1 or 24h to the MTC of each metal. The MTC cultures were obtained by continuously culturing cells at their maximum tolerated metal concentration. To study the persistence of the metal adaptation process, we grew the metal adapted strains for 1 or 6 months in PP210 medium in the absence of added metal. Following these periods, cells were again incubated for 1 or 24h in the presence of the MTC of each metal.

### Total RNA isolation and cDNA synthesis

Cultures (1–3 x 10^5^ cells/ml) of the different *T*. *thermophila* strains were harvested by centrifugation at 2,800 rpm for 3 min. Total RNA samples were isolated from exponential cell cultures by using the TRI Reagent method (Molecular Research Center, MRC). RNA samples were treated with DNase I (Roche) for 30 min at 37°C and visualized following agarose gel electrophoresis. RNA concentrations were determined using NanoDrop 1000 (Thermo Scientific). MultiScribe Reverse Transcriptase 50 units/μl (Life Technologies) and oligo(dT)-adaptor primer (Roche) were used to synthesize the cDNAs from 3.5 μg of total RNA samples.

### Quantitative RT-PCR (qRT-PCR)

cDNA samples were amplified in duplicate in 96 microtiter plates. Each qPCR reaction (20 μl) contained: 10 μl of SBYR Green (Takara), 0.4 μl of ROX as passive reference dye (Takara), 1 μl of each primer (at 400 nM final concentration), 3.6 μl of ultrapure sterile water (Roche) and 4 μl of a 10^−1^ dilution of cDNA. PCR primers (S1 Table) were designed using the "Primer Quest and Probe Design" online-application of IDT (Integrated DNA Technologies). β-actin was used as an endogenous control or a normalizer gene. Melting curves were obtained and primers specificity was tested by confirming each PCR product by gel electrophoresis and sequencing. Real-time PCR reactions were carried out in an iQ5 real-time PCR apparatus (Bio-Rad) and the thermal cycling protocol was as follows: 5 min at 95°C, 40 cycles (30 sec at 95°C, 30 sec at 55°C and 20 sec at 72°C), 1 min at 95°C and 1 min at 55°C. All controls (no template control and RT minus control) were negative. Amplification efficiency (E) was measured by using 10-fold serial dilutions of a positive control PCR template. The efficiency requirement was met for all the tested genes in all the used strains (S2 Table). Results were finally processed by the standard-curve method [39] and were corroborated with at least two independent experiments, each performed in duplicate. We compared the basal expression levels of different genes using the formula: 2^(Ct1-Ct2)^, being C_t1_ and C_t2_ the cycle threshold (C_t_) values of both genes under a control situation (no metal exposure).

### Statistical analysis

Gene expression differences were tested for statistical significance by Student’s t test using the program Statgraphics Centurion XVI (16.1.15 version). P-value was fixed in ≤ 0.05.

## Results

### Comparative MT gene expression analysis among different strains of *T*. *thermophila*

Over a period of more than two years, we adapted three *T*. *thermophila* cultures to high concentrations of metals. These were named Cd-adap, Cu-adap and Pb-adap. The maximum tolerated concentrations (MCT) were 115 μM Cd^2+^, 4 mM Cu^2+^, and 5.5 mM Pb^2+^. These MTC values are substantially higher than the LC_50_ values (lethal concentration killing 50% of cell population) previously determined for the parental SB1969 strain in PP210 medium (24 h exposure): ≈ 2.5x the LC_50_ for Cd^2+^ (44.5 μM), ≈ 12.7x the LC_50_ for Cu^2+^ (315 μM), and ≈ 5.7x the LC_50_ for Pb^2+^ (965 μM) [20]. In the course of constructing KO strains, we found that it was impossible to disrupt all macronuclear copies of *MTT5*, indicating that the gene is essential. Therefore, the strain that we obtained should be considered a knockdown (MTT5KD). In total, we obtained a MTT1KO strain, a double mutant MTT1KO+MTT5KD, and the MTT5KD strain.

We compared the gene expression of *MTT1*, *MTT3*, *MTT5* and *MTT2/MTT4* between different *T*. *thermophila* strains; the SB1969 control strain, the three metal-adapted strains (Cd-adap, Cu-adap and Pb-adap), the three knockout (KO) and/or knockdown (KD) strains described above, and GFPMTT1 and GFPMTT5 that over-express *MTT1* or *MTT5*, respectively [36]. *MTT2* and *MTT4* are 98% identical [20] so it is not possible to design specific primers to distinguish them by qRT-PCR. Therefore, we refer to them collectively as *MTT2/4*, because we evaluated the expression of both genes together using primers *MTT2QA* and *MTT2QB* (S1 Table).

**Fig 2:**
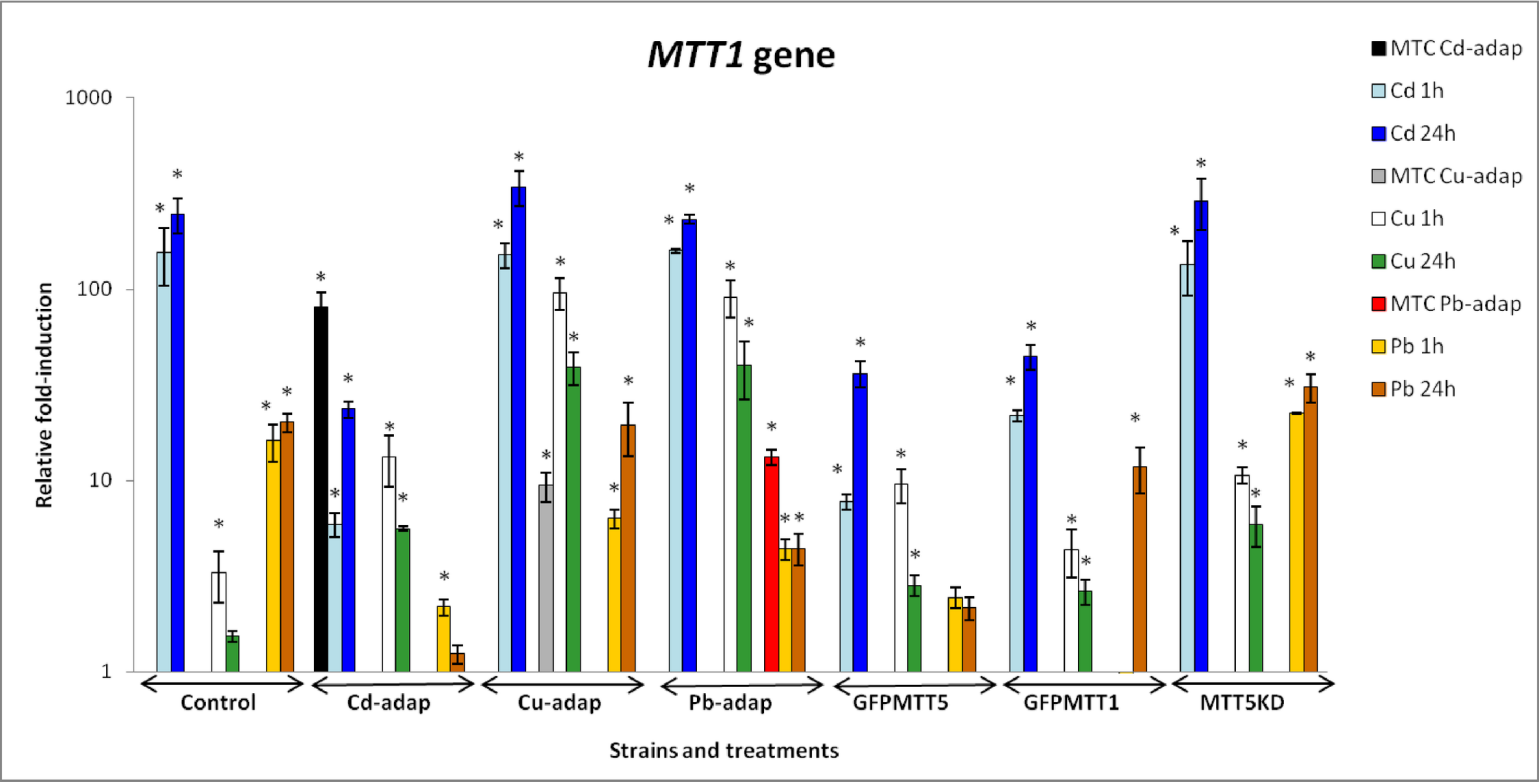
Comparison of relative fold-induction of *MTT1* in different *T*. *thermophila* strains. MTC: maximum tolerated concentration (115 μM Cd^2+^, 4 mM Cu^2+^ or 5.5 mM Pb^2+^). β-actin was used as the normalizer gene. Each histogram bar represents an average value ± standard deviation (see S3 Table) from two or three independent experiments. Relative induction values are represented in a logarithmic scale. Asterisks indicate significant differences from the control with p ≤ 0.05.

Relative fold-induction values for *MTT1* are shown in Fig 2 and S3 Table. *MTT1* responds preferably to Cd^2+^ in all studied strains and, in general, 24h Cd^2+^ or Pb^2+^ exposures result in higher relative induction than 1h. However, the opposite effect is observed for Cu^2+^, which induces a stronger *MTT1* induction after 1h than after 24h (Fig 2). Moreover, in Cd-adap and Pb-adap strains, *MTT1* induction of MTC cultures were higher than those after 1 or 24h treatments. On the other hand, in the Cu-adap strain the MTC culture induced *MTT1* less strongly than in cultures treated for 1 or 24h (Fig 2). In general, *MTT1* fold-induction values from control, GFPMTT1 and MTT5KD strains show the following ranking: Cd > Pb > Cu. For Cd-adap, Cu-adap, Pb-adap and GFPMTT5 strains, this ranking changes to: Cd > Cu > Pb (Fig 2). *MTT1* is not induced (fold-induction < 2) in GFPMTT1 after 1h Pb^2+^ treatment, but it is induced (around 11-fold) in this strain after 24h treatment at the same Pb^2+^ concentration. Likewise, this gene is not induced in control and Cd-adap strains at 24h Cu^2+^ or Pb^2+^ treatments (Fig 2, S3 Table).

**Fig 3:**
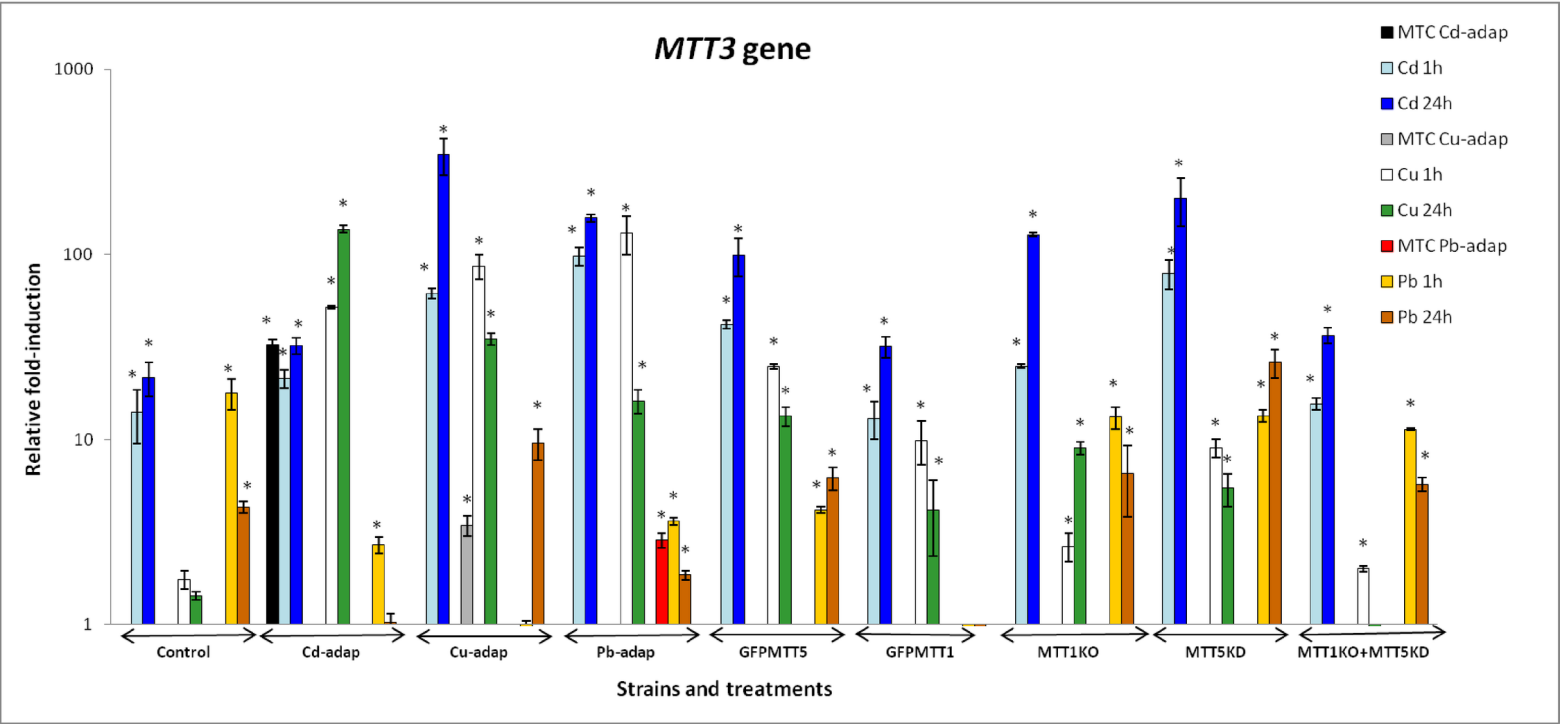
Comparison of relative fold-induction of *MTT3* in different *T*. *thermophila* strains. MTC: maximum tolerated concentration (115 μM Cd^2+^, 4 mM Cu^2+^ or 5.5 mM Pb^2+^). β-actin was used as the normalizer gene. Each histogram bar represents an average value ± standard deviation (see S3 Table) from two or three independent experiments. Relative induction values are represented in a logarithmic scale. Asterisks indicate significant differences from the control with p ≤ 0.05.

Relative fold-induction for *MTT3* is reported in Fig 3 and S3 Table. Like *MTT1*, *MTT3* preferentially responds to Cd^2+^ in almost all strains. The exception is the Cd-adap strain, in which stronger induction occurs after Cu^2+^ treatments than after Cd^2+^ (Fig 3). In general, 24h Cd^2+^ treatments cause higher relative *MTT3* induction than after 1h, while for Cu^2+^ or Pb^2+^ treatments the highest induction depends on the strain rather than the metal exposure time. In Cd-adap and Pb-adap strains, the induction of *MTT3* in MTC cultures was very similar to that obtained after 1 or 24h. In contrast, in the Cu-adap strain, *MTT3* induction in MTC cultures was lower than after 1 or 24h (Fig 3). In control, MTT1KO, MTT5KD and MTT1KO+MTT5KD strains, *MTT3* shows the following fold-induction ranking: Cd > Pb > Cu, whereas in Cu-adap, Pb-adap, GFPMTT5 and GFPMTT1 strains the ranking for *MTT3* is: Cd > Cu > Pb. Finally, for the Cd-adap strain, *MTT3* induction shows the following ranking: Cu > Cd > Pb (Fig 3). Following 1h of Cd^2+^ exposure, *MTT3* gene induction values in MTT1KO and MTT5KD were considerably larger than in control strains (≈ 2- and ≈ 6-fold, respectively), and the same was true after 24h exposure (≈ 6- and ≈ 9-fold, respectively). However, in the MTT1KO + MTT5KD strain, *MTT3* maintains similar induction values after Cd^2+^ exposures to those seen in control strain (Fig 3, S3 Table). Finally, *MTT3* showed no induction in the following cases: in GFPMTT1 after 1 or 24h Pb^2+^ treatment; in the control strain after 1 or 24h Cu^2+^ treatments; in the Cd-adap (24h Pb^2+^); in the Cu-adap (1h Pb^2+^) and; in the MTT1KO + MTT5KD strain (24h Cu^2+^) (Fig 3, S3 Table).

**Fig 4:**
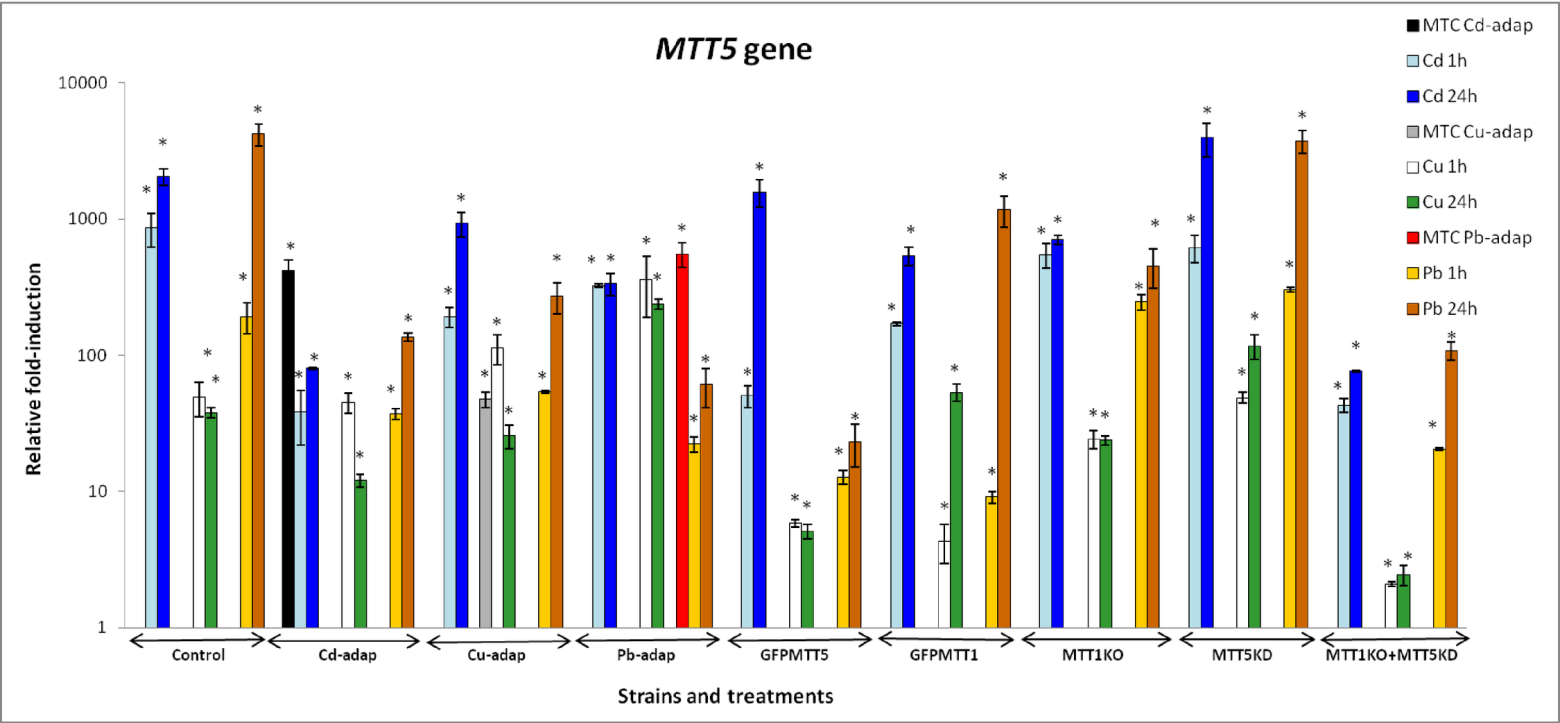
Comparison of relative fold-induction of *MTT5* in different *T*. *thermophila* strains. MTC: maximum tolerated concentration (115 μM Cd^2+^, 4 mM Cu^2+^ or 5.5 mM Pb^2+^). β-actin was used as the normalizer gene. Each histogram bar represents an average value ± standard deviation (see S3 Table) from two or three independent experiments. Relative induction values are represented in a logarithmic scale. Asterisks indicate significant differences from the control with p ≤ 0.05.

*MTT5* shows the highest fold-induction among the five *T*. *thermophila* MT genes, independent of the analyzed strain. Unlike *MTT1* and *MTT3*, it is induced under all the assayed metal treatments in all strains (S3 Table). *MTT5* preferentially responds to Cd^2+^ and Pb^2+^ and shows strongest induction after 24h treatments (Fig 4, S3 Table). In the majority of the strains, the *MTT5* fold-induction ranking is: Pb ≥ Cd > Cu. The exception is the Pb-adap strain, where the ranking is: Cd = Cu ≥ Pb (Fig 4). In metal-adapted strains, the MTC cultures have higher *MTT5* induction values than those reported at 1 or 24h for Cd^2+^ and Pb^2+^ exposures, while for Cu^2+^ treatments the gene induction values in MTC cultures are lower than after 1h treatments. After Cd^2+^ treatments (1 or 24h), the MTT1KO and the MTT1KO + MTT5KD strains have lower *MTT5* induction values than the control strain, while these values are higher (≈ 2x) in the MTT5KD strain after 24h Cd^2+^ treatments, compared to the control strain. After 1h Pb^2+^ exposures, MTT1KO and MTT5KD have higher *MTT5* induction values than the control strain, while after 24h Pb^2+^ treatments these values are lower (in MTT1KO) or similar (in MTT5KD strain) relative to the control strain (Fig 4, S3 Table).

**Fig 5:**
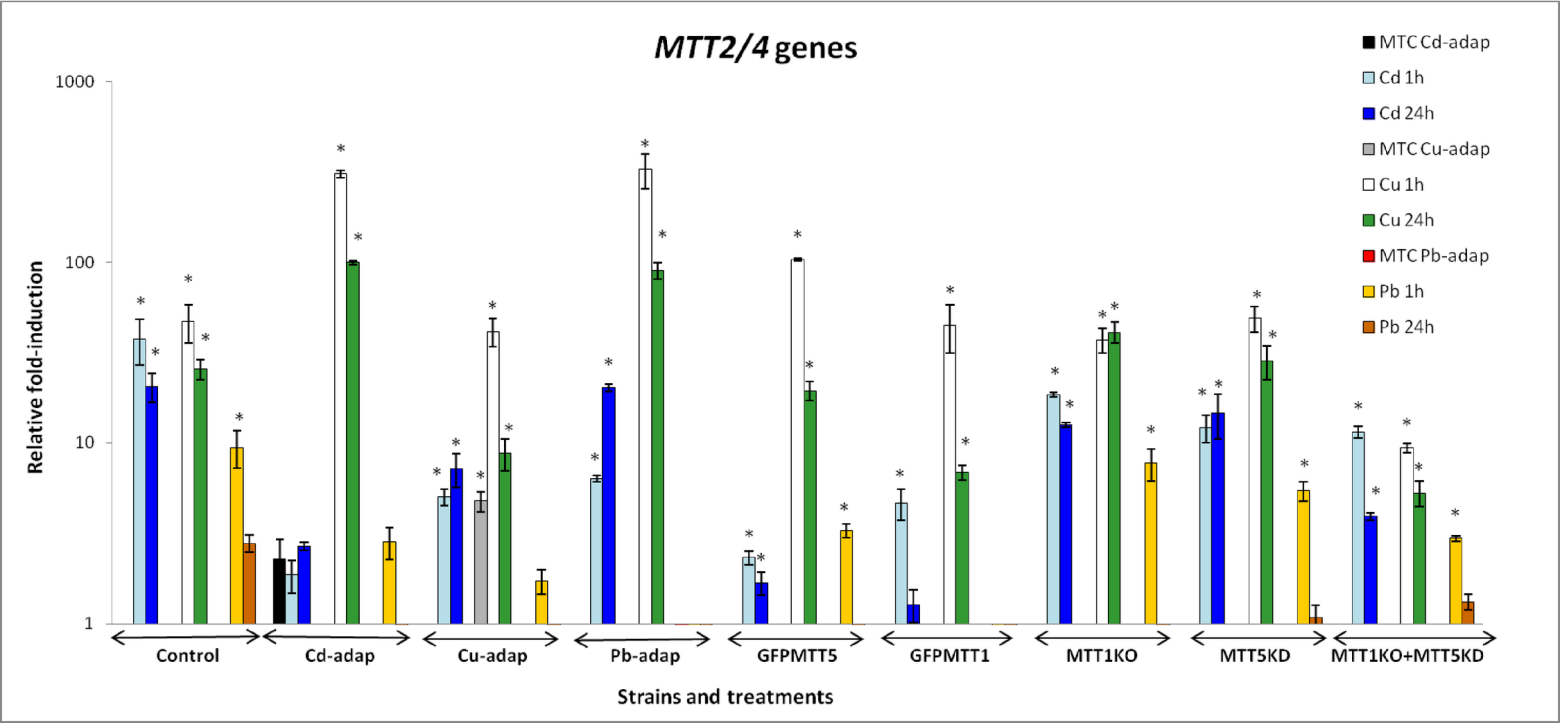
Comparison of relative fold-induction of *MTT2/4* in different *T*. *thermophila* strains. MTC: maximum tolerated concentration (115 μM Cd^2+^, 4 mM Cu^2+^ or 5.5 mM Pb^2+^). β-actin was used as the normalizer gene. Each histogram bar represents an average value ± standard deviation (see S3 Table) from two or three independent experiments. Relative induction values are represented in a logarithmic scale. Asterisks indicate significant differences from the control with p ≤ 0.05.

*MTT2/4* responds most strongly to Cu^2+^, showing the highest fold-induction values after 1h treatment with this metal in all strains (Fig 5). These genes are also significantly induced upon Cd^2+^ exposures in most strains, but do not respond to 24h Pb^2+^ treatments. Moreover, *MTT2/4* are only significantly induced after 1h Pb^2+^ exposures in control, GFPMTT5, MTT1KO, MTT5KD and MTT1KO + MTT5KD strains. In all strains the fold- induction values for *MTT2/4* follow this ranking: Cu > Cd > Pb. MTT1KO and MTT5KD strains show similar *MTT2/4* induction to the control under Cu^2+^ stress. However, the MTT1KO + MTT5KD strain has considerably weaker *MTT2/4* induction than the control strain (S3 Table). *MTT2/4* are not induced in the following strains and treatments: control strain (24h Pb^2+^); Cd-adap (all Cd^2+^ and Pb^2+^ exposures); Cu-adap (1 and 24h Pb^2+^); Pb-adap and GFPMTT1 strains (all Pb^2+^ exposures); GFPMTT5, MTT1KO, MTT5KD and MTT1KO + MTT5KD strains (1h Pb^2+^). Therefore, *MTT2/4* show the weakest induction among the MT genes, under the conditions tested (Fig 5, S3 Table).

### Comparative MT gene expression analysis of metal-adapted strains from reversible adaptive metal resistance experiments

The three metal-adapted strains, generated as described in Materials and Methods, were transferred and maintained for 1 or 6 months in PP210 without any added metal (indicated as -1M or -6M strains). Afterward, they were again exposed to the MTC of the metal to which they had been adapted, for 1 or 24h, and MT gene induction was measured. The three *T*. *thermophila* pre-adapted strains were able to survive in the presence of the relevant metal MTC, and no significant cell mortality was detected by microscopy.

**Fig 6:**
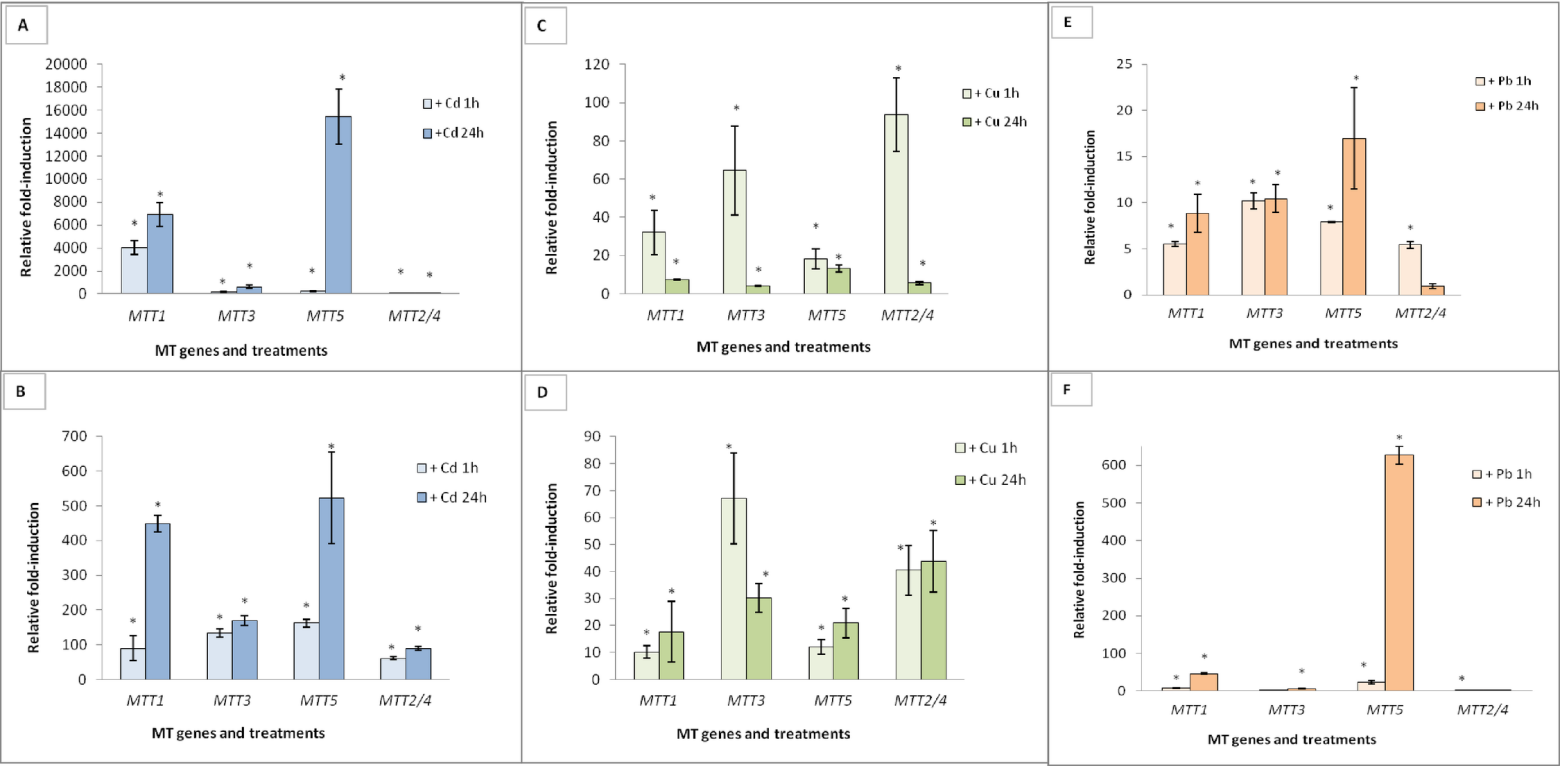
Relative fold-induction of the five *T*. *thermophila* MT genes in metal-adapted strains. These strains were maintained for 1 or 6 months without the metal treatment and then re-exposed (1 or 24h) to the relevant MTC. (A): Cd-adap after 1 month without metal (-1M). (B): Cd-adap after 6 months without metal (-6M). (C): Cu-adap (-1M). (D): Cu-adap (-6M). (E): Pb-adap (-1M). (F): Pb-adap (-6M). β-actin was used as the normalizer gene. Each histogram bar represents an average value ± standard deviation (see S3 Table) from two or three independent experiments. Asterisks indicate significant differences from the control with p ≤ 0.05.

Gene induction values are shown in Fig 6. The Cd-adap strain maintained the same ranking of relative gene induction after 1 or 6 months of culture in the absence of Cd^2+^: *MTT5* > *MTT1* > *MTT3* > *MTT2/4* (Fig 6A, S3 Table). However, the absolute induction levels in the -1M culture were higher than seen previously, most notably for *MTT5* and *MTT1* (Fig 6A, S3 Table). The fold-induction decreased in the -6M sample, compared to -1M, but remained higher than the induction seen in the founder Cd-adap culture (Fig 6B). For *MTT1*, *MTT3* and *MTT5*, 24h Cd^2+^ addition to -1M or -6M cultures generated higher fold-induction values than 1h addition. For *MTT2/4*, the highest expression levels were achieved after 1h Cd^2+^ treatments (Fig 6).

For the Cu-adap strain, the induction ranking of the founder culture was *MTT5 > MTT1 >MTT3 >MTT2/4*, as described above. This induction ranking changed considerably, after 1 month culture in medium lacking added Cu^2+^, to (*MTT2/4 > MTT3 > MTT1 > MTT5*), and again after 6 months to (*MTT3 > MTT2/4 > MTT5 ≈ MTT1*) (Fig 6C and 6D). In the Cu-adap (-1M) culture, induction (mainly for *MTT2/4* and *MTT3*) is considerably higher after 1h Cu^2+^ treatments than after 24h Cu^2+^ or than induction in the founder Cu-adap strain (MTC samples) (Fig 6C, S3 Table). However, the Cu-adap (-6M) culture generally showed stronger induction after 24h treatment than after 1h, the exception being *MTT3* (Fig 6D).

For the Pb-adap strain, the induction ranking of the founder culture was *MTT5 > MTT1 > MTT3 > MTT2/4*, as described above. The Pb-adap (-1M) and (-6M) strains maintained a very similar induction ranking (*MTT5 > MTT1 ≥ MTT3 > MTT2/4*) (Fig 6E and 6F, S3 Table). In the -1M culture, *MTT1*, *MTT3* and *MTT2/4* induction values were higher than those in the founder culture; however, *MTT5* induction was lower than the in the founder (Fig 6E, S3 Table). In the -6M culture, *MTT5* was enormously induced after 24h Pb^2+^ exposure, similar to that seen in the founder culture (S3 Table). *MTT1* also responded under these conditions, mainly after 24h Pb^2+^ exposure, while *MTT2/4* and *MTT3* showed less induction than in the Pb-adap (-1M) strain (Fig 6E and 6F). In general, 24h treatments resulted in similar or stronger induction compared to induction after 1h metal exposure, for Pb-adap (-1M) and (-6M) cultures (Fig 6E and 6F).

### Comparative analysis of constitutive MT gene expression levels among the different *T*. *thermophila* strains

*T*. *thermophila* MT genes show constitutive expression in wildtype cells under non-stressful conditions [20]. For the strains generated in the present study, we compared constitutive (basal) expression levels using the C_t_ values obtained in the absence of added metal.

In qPCR (quantitative polymerase chain reaction) or qRT-PCR studies, the threshold is defined as the level of signal that reflects a statistically significant increase over the calculated baseline signal. It is set to distinguish a relevant amplification signal from the background. The threshold cycle (C_t_) value is the cycle number at which the fluorescent signal of the reaction crosses the threshold, and is inversely related to the amount of starting template. For example, low C_t_ values correspond with high levels of transcripts (qRT-PCR templates) or DNA copy number (qPCR templates). The C_t_ values corresponding to basal gene expression levels are reported in S4 Table. We used these data to compare basal C_t_ values of different MT genes within the same strain (S5 Table), or of individual MT genes between the different *T*. *thermophila* strains (S6 Table). In particular, we calculated 2 ^(Ct1-Ct2)^, where C_t1_ and C_t2_ are the C_t_ values of two different samples.

As shown in S4 Table and S1 Fig, *MTT1* has the highest basal expression in most strains (SB1969, Cd-adap, Pb-adap, GFPMTT1, GFPMTT5, MTT5KD, -1M and - 6M metal-adapted cultures). *MTT2/4* shows highest basal expression in Cu-adap, Cu-adap (-1M) and MTT1KO strains. *MTT5* shows the highest basal values only in the MTT1KO + MTT5KD and Pb-adap (-1M) culture. Therefore, in general, *MTT5* and *MTT3* have the lowest basal expression, ranked third or fourth in most strains (SB1969, Cd-adap, Cu-adap, Cd-adap (-1M and -6M), Cu-adap (-1M and -6M), GFPMTT1, MTT1KO and MTT5KD) (S1 Fig).

The MT basal expression ranking in the SB1969 control strain is *MTT1* > *MTT3* > *MTT2/4* > *MTT5*. The absolute level of *MTT1* expression is about 1.6x *MTT3*, ≈ 8x *MTT2/4* and ≈ 36x *MTT5*. For *MTT3*, expression is about 5x *MTT2/4* and 22x *MTT5*. *MTT2/4* expression is ≈ 4x *MTT5* (S5 Table). In the Cd-adap strain, *MTT1* ≈ 30x *MTT3*, ≈ 68x *MTT5* and ≈118x *MTT2/4*, while *MTT3* ≈ 2x *MTT5* and ≈ 4x *MTT2/4*. *MTT5 expression* ≈ 2x *MTT2/4* (S5 Table). While *MTT1* in this Cd-adap culture, like in the control strain, shows the highest basal expression, the differences between basal expression levels of *MTT1* vs the other MT genes are greater in the adapted culture: 2-fold for *MTT5*, ≈ 15-fold for *MTT2/4*, and ≈19-fold for *MTT3* (S5 Table). *MTT2/4* showed highest basal expression levels in the Cu-adap strain, being ≈ 27x *MTT5* and 16x *MTT3*. For the Cu-adap (-1M) culture, *MTT2/4* = ≈ 11x *MTT5* and 6.5x *MTT3* (S5 Table). *MTT1* and *MTT2/4* show similar high basal expression. The ranking of relative expression levels is modified in the Cu-adap (- 6M) culture: *MTT1* > *MTT2/4* > *MTT5* > *MTT3* (S1 Fig, S5 Table). *MTT1* is ranked first for basal expression in the Pb-adap and Pb-adap (-6M) cultures, but this gene is ranked second in the Pb-adap (-1M) culture (*MTT5* > *MTT1* > *MTT2/4* > *MTT3*) (S1 Fig, S5 Table).

In the GFPMTT1 and GFPMTT5 strains, *MTT1* also shows the highest basal expression levels, with the following rank: *MTT1* ≈ 35x *MTT5* and 4x *MTT3* in GFPMTT1 and ≈ 3x *MTT5* and 22x *MTT3* in GFPMTT5 (S5 Table). This last ratio (*MTT1* vs. *MTT3*) is considerably higher than that in SB1969 (S5 Table). In MTT1KO and MTT1KO + MTT5KD strains, *MTT2/4* and *MTT5* (respectively) show the highest basal expression levels (S1 Fig). The ranking in MTT1KO is *MTT2/4* ≈ 4x *MTT5*. For MTT1KO + MTT5KD, the ranking is *MTT5* ≈ 6x MTT3 and *MTT2/4* ≈ 4.6x *MTT3* (S5 Table). For MTT5KD, *MTT1* has the highest basal expression level, and *MTT1* ≈ 2x *MTT5* and 34x *MTT3* (S5 Table).

Overall, the highest basal expression for *MTT1* and *MTT3* are in the Cd-adap culture, while *MTT2/4* are expressed most strongly in the Cu-adap culture. *MTT5* is most strongly expressed in GFPMTT5 (S6 Table). We also compared the basal expression levels to those in the control strain. *MTT2/4* expression levels are considerably increased in GFPMTT1 (≈ 7x control). In GFPMTT5, the basal expression levels of *MTT5* is increased ≈ 64x compared to the control strain, and the corresponding increases for *MTT1* ≈ 4.5x, and for *MTT2/4* ≈ 6.5x (S6 Table).

Similarly, in the metal-adapted cultures *MTT1* and *MTT5* genes show increased basal expression; ≈ 28x and 15x, respectively, in the Cd-adap strain, while *MTT2/4* basal expression is augmented ≈ 17.5x in Cu-adap strain. *MTT5* shows increased basal expression in the Pb-adap strain (≈ 7.5x) (S6 Table).

Several changes occurred in basal expression of the metal-adapted strains after they were maintained for 1 or 6 months without added metal. *MTT1* expression returned to the level of the control strain in the Cd-adap (-1M) culture, but the same cells maintained higher basal expression of *MTT2/4* (≈ 4x) and *MTT5* (≈ 6x) relative to the control. This higher *MTT5* basal expression is also maintained in the Cd-adap (-6M) strain (S6 Table).

*MTT2/4* basal expression was considerably reduced in the Cu-adap (-1M) culture, but are nonetheless still higher (≈ 4x) compared to the control strain. *MTT2/4* expression returned to control levels in the Cu-adap (-6M) strain, but there was an increase in *MTT5* basal expression (≈ 8.5x) (S6 Table). *MTT5* basal expression increased more dramatically in Pb-adap (-1M) and (- 6M), to ≈ 168x and 21x the control strain levels (S6 Table). Finally, the MTT1KO and MTT1KO + MTT5KD strains both showed increased basal expression compared to the control strain of *MTT5* (≈ 8.5x and ≈ 60x, respectively) and *MTT2/4* (≈ 8x and ≈ 10x, respectively) (S6 Table). The basal expression ranking in MTT5KD was similar to that of GFPMTT1 (S1 Fig).

## Discussion

In the work described in this manuscript, we have focused on a set of unanswered questions regarding the regulation and specific functions of the individual *T*. *thermophila* MT isoforms. By comparing *T*. *thermophila* MT gene expression under different metal stresses and using the metal-adapted (Cd-adap, Cu-adap and Pb-adap), knockout and/or knockdown (MTT1KO, MTT5KD and MTT1 + MTT5KD) and other strains (SB1969 control, GFPMTT1 and GFPMTT5), we obtained a better understanding of the specific roles of each MT isoform.

One approach we have taken is to adapt *Tetrahymena* cultures over extended periods to high metal concentrations. Such adaptation programs, in which organisms are increasingly exposed to a specific external stress, can reveal the cellular mechanisms and pathways that underlie the normal stress response. In such an experimental evolution experiment, the continuous adaptation to a specific stress can serve to magnify and reveal cellular efforts to counteract any harmful effects. One mechanism of adaptation is modulation of gene expression ("gene expression plasticity"), which can produce new adaptive phenotypes in the face of a specific stress [40].

In our experiments, different *T*. *thermophila* cultures were cultured in the presence of increasing metal (Cd^2+^, Cu^2+^ or Pb^2+^) concentrations. We could thus define a maximum tolerated concentration (MTC). These MTC values were considerably higher than the corresponding LC_50_ values in this species previously determined for each metal [20]. The relative increase in MTC values (i.e., between the adapted vs non adapted cultures) was inversely correlated with the toxicity of the specific metals: Cu^2+^ (≈ 12.7x) > Pb^2+^ (≈ 5.7x) > Cd^2+^ (≈ 2.5x). We studied cultures that were maintained at their respective MTCs (Cd-adap, Cu-adap or Pb-adap) for extended periods.

### The *MTT1* isoform: A gene with a relevant and still unknown constitutive function

*MTT1* is most strongly induced by Cd^2+^ in all strains, as previously reported in the wild-type 20]. In addition, the MTT1 protein showed the highest affinity for Cd^2+^ among the *T*. *thermophila* CdMTs [27]. However, *MTT1* also responded to Cu^2+^ or Pb^2+^, with different expression patterns depending on the metal. With Cd^2+^ or Pb^2+^, *MTT1* was induced by 1h but the highest induction values were achieved after 24h. With Cu^2+^, the highest *MTT1* induction occurred after 1h. A difference in induction by Cd^2+^/Pb^2+^ vs Cu^2+^ was also observed in the metal-adapted strains. Both Cd-adap and Pb-adap strains showed strongest *MTT1* induction under continuous exposure to the MTC, corroborating that the persistent presence of these metals is required to maintain highest *MTT1* expression. In contrast, the strongest *MTT1* induction in the Cu-adap strain occurred after growth for 24h without any added metal, and then re-exposing the cells to Cu^2+^ for 1h.

Among the *T*. *thermophila* MT proteins, MTT1 shows the lowest affinity for Cu^2+^ [27] and the *MTT1* gene shows weakest induction by this metal. Unlike Cd^2+^ or Pb^2+^, Cu^2+^ is an essential metal and is comparatively less toxic, which may account for the difference in the *MTT1* transcriptional response to this metal. *MTT1* expression occurred soon after exposure to Cu^2+^, and then decreased over time. In contrast, the induction in response to Cd^2+^ or Pb^2+^ was more persistent. These metals may be increasingly toxic with long exposure, and therefore require more prolonged strong *MTT1* induction. A similar disparity between the effects of Cd^2+^ or Cu^2+^ stress on transcription was reported for *MTT3*, *MTT5* and *MTT2/4*, as well as on MT gene transcription of other *Tetrahymena* species [6]. In general, two different MT gene expression patterns can be distinguished in *Tetrahymena*. In response to Cu^2+^, the high initial transcript levels subsequently decrease over time. In contrast, the transcriptional response to Cd^2+^ or Pb^2+^ increases over time. These differences in the transcriptional responses are correlated with the essential vs. non-essential nature of the metals, and their levels of toxicity, but the underlying molecular or physiological mechanisms are not yet known.

*MTT1* constitutive expression differed significantly between strains, with the following relative ranking: Cd-adap > GFPMTT5 > GFPMTT1 > Control strain > Cu-adap ≈ Pb-adap > MTT5KD. Compared to the control, expression was ≈ 28x higher in Cd-adap, ≈ 5x higher in GFPMTT5 and 2x higher in GFPMTT1. This is probably the reason why *MTT1* gene induction levels in the GFPMTT1 strain were not higher than those in the control strain (Fig 2). Based on these results, we can conclude the following points. First, Cd-adaptation involves an increase of basal *MTT1* expression. Similar phenomena have been reported in other organisms. For instance, an increase in constitutive MT expression plays an important function when the arthropod *Orchesella cincta* is chronically exposed to Cd^2+^ [41]. Likewise, in the domestic fly (*Musca domestica*), a cytochrome P450 isoform that confers insecticide resistance shows 9-fold higher constitutive expression in a resistant strain compared to a non-resistant one [42]. In some freshwater snail species, like *Biomphalaria glabrata*, constitutive expression of a MT gene seems to confer tolerance to the snails against Cd^2+^ exposure [43].

GFPMTT1 contains multiple copies of the recombinant plasmid pVGFMTT1 with the construct *P*_*MTT1*_::*GFP*::*MTT1* (complete *MTT1* ORF under its own *MTT1* promoter) [36]. Therefore, *MTT1* is over-expressed, including the expected increase in basal expression. In GFPMTT5, *MTT5* is driven by the *MTT1* promoter and its constitutive expression levels are ≈ 49x higher than in the control. The *MTT1* promoter driving the heterologous construct is more active under basal conditions than the endogenous *MTT5* promoter, accounting for the observed increase in *MTT5* basal expression. Interestingly, *MTT1* basal expression is also considerably higher in this strain compared to the wildtype (≈ 5x) and to the GFPMTT1 strain (≈ 2x). In conclusion, Cd-adap, GFPMTT1 and GFPMTT5 show increased *MTT1* basal expression. This increase can be explained by a greater *MTT1* copy number in GFPMTT1, and this may also be true in the Cd-adap strain (unreported results).

**Fig 7:**
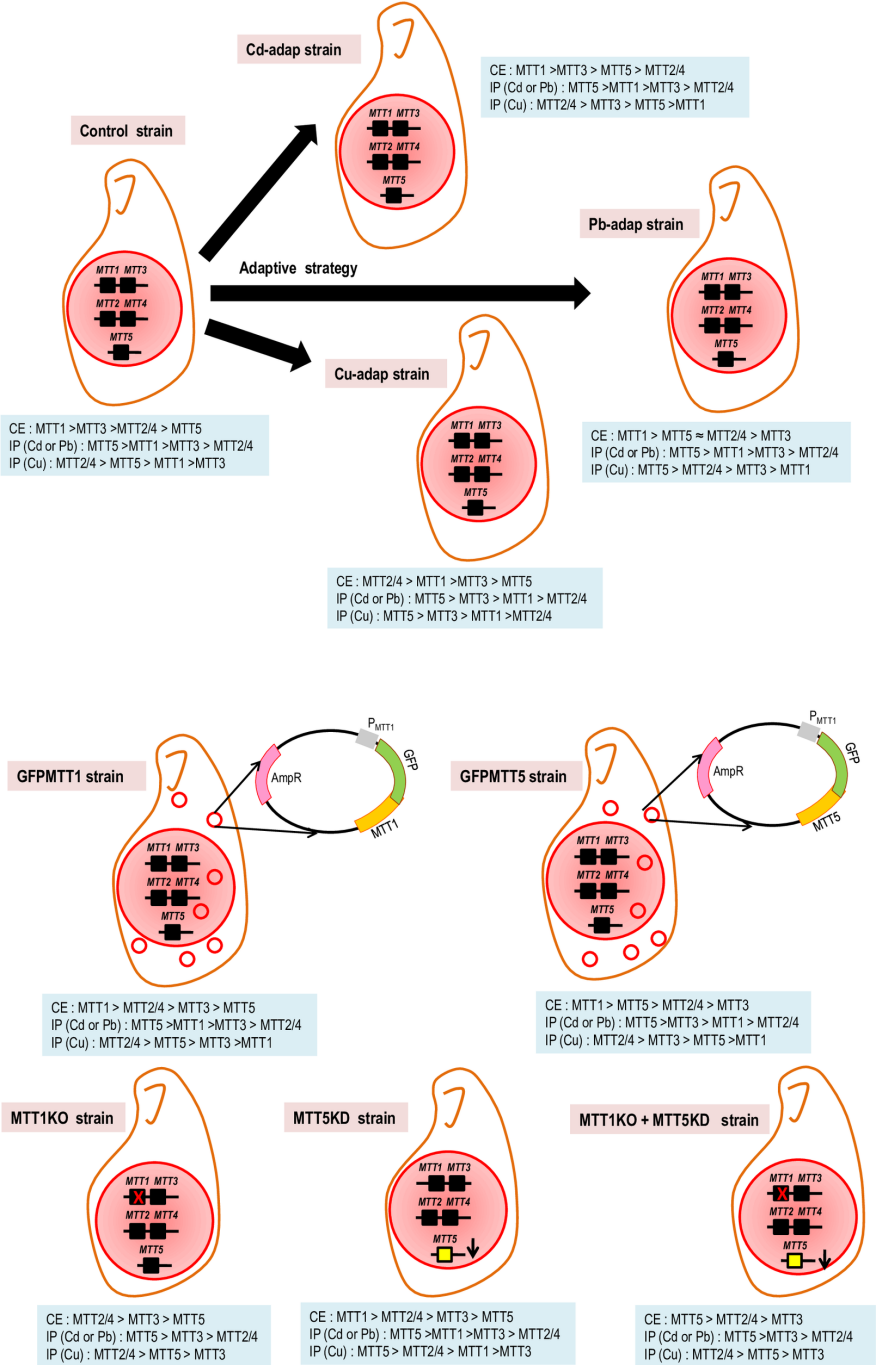
Schematic representation of the genomic characteristics and the MT gene expression levels from the different *T*. *thermophila* strains used in this study. The constitutive MT gene expression ranking (CE) and the MT gene induction patterns (IP) obtained under Cd^2+^/ Pb^2+^ or Cu^2+^ treatments are shown for each strain.

In GFPMTT5, the increase of *MTT1* basal expression may be due to higher *MTT5* basal expression driven by the *MTT1* promoter. This can be explained if basal expression is coordinated between MT genes, in this case *MTT1* and *MTT5*. Van Straalen et al. [44] have pointed out that both *cis* and *trans*-regulatory mechanisms can contribute, in a combinatorial fashion, to adaptive evolution in response to a stress. We detected more hints of coordinated gene regulation in the knockout (KO) and/or knockdown (KD) strains. MTT1KO and MTT1KO + MTT5KD, lacking all copies of *MTT1*, show increased *MTT5* basal expression (≈ 8.5x control for MTT1KO and ≈ 59x for MTT1KO + MTT5KD). *MTT5* may be upregulated to compensate for the absent *MTT1* activity in these strains. It appears that while *MTT1* is not essential, its activity is important in stress but also non-stress conditions, where basal expression may contribute to cellular homeostasis. Consistent with this idea, basal *MTT1* expression is the highest among the MT isoforms in all strains except the Cu-adap culture (S5 Table). The ranking of MT basal expression in GFPMTT1 is *MTT1* > *MTT2/4* > *MTT3* > *MTT5*, which is very similar to the control (*MTT1* > *MTT3* > *MTT2/4* > *MTT5*). However, in the GFPMTT5 strain, *MTT5* is second in basal expression (*MTT1* > *MTT5* > *MTT2/4* > *MTT3*), and a similar ranking was found for the Pb-adap strain (*MTT1* > *MTT5* > *MTT2/4* > MTT3) (Fig 7, S5 Table).

### The *MTT5* isoform: An essential "alarm" MT gene

Based on previous studies, *MTT5* was considered the main MT gene involved in metal detoxification [5, 26]. This protein represents one of the best Cd-thionein after MTT1 due to its high affinity for this metal cation [27]. These conclusions are extended by our present results. *MTT5* is the most strongly induced MT gene for any metal treatment and in all strains. The consensus MT gene induction pattern following exposure to Cd^2+^ or Pb^2+^ is *MTT5* > *MTT1* / *MTT3* > *MTT2/4* (Fig 7). This includes MTT5KD which has a considerably reduced *MTT5* copy number (unreported results). Moreover, although the *MTT5* gene product has a low affinity for Cu^2+^ [27], it is the most highly induced MT following Cu^2+^ treatment in three strains (Cu-adap, Pb-adap and MTT5KD) (Fig 7). This strong induction under different metal stress treatments may be explained by the presence of a 416 bp duplicated sequence in its promoter region [20], which includes more putative binding sites for AP-1 transcription factors than are found in other *T*. *thermophila* MT isoforms [5, 6, 20, 26].

Basal expression of *MTT5* is much lower than that of other MTs in several strains (control, Cu-adap, GFPMTT1, MTT1KO and MTT5KD) (Fig 7). In SB1969, basal expression of *MTT1*, *MTT3* and *MTT2/4* are ≈ 36x, 23x, and 4x the expression of MTT5, respectively. In Cd-adap and Pb-adap strains, *MTT5* is ranked third and second in basal expression, respectively (Fig 7). However, *MTT5* is the most highly expressed MT in the MTT1KO + MTT5KD strain, where *MTT1* is absent and *MTT5* gene copy number is drastically reduced (Fig 7). Basal *MTT5* expression in MTT1KO is ≈1,000x that in the MTT5KD strain, a difference which is due to the reduced *MTT5* copy number in MTT5KD and likely compensation in MTT1KO for the absence of *MTT1*. Taken together, our data suggest that the balance of the *MTT5* and *MTT1* activities may regulate the expression of both genes.

Unlike *MTT1*, which shows high basal expression, *MTT5* does not seem to be required in non-stress conditions, judging by its low basal expression. Expression increases very quickly, to high levels, under stress conditions. In this sense, *MTT5* might be considered an "alarm” MT gene, while at the same time it is essential for cell viability. As an essential gene, *MTT5* may be required to induce other MT isoforms (e.g., MTT1 or MTT2/4), which may be needed depending on the specific stressor and the total available MT pool. GFPMTT5 shows ≈ 49-fold increased basal expression for this "alarm” MT gene. This might be interpreted by these cells as a stress situation, accounting for the increased expression of other MTs: a ≈ 5x increase for *MTT1* and ≈ 6.5x increase for *MTT2/4*. The MTT1KO + MTT5KD strain must respond to a distinct genetic stress: loss of *MTT1* and drastically reduced *MTT5* copy number. Facing this situation, this strain shows ≈ 59x over-expression of *MTT5* and ≈ 10x over-expression of *MTT2/4*. These results are consistent with the idea that expression of *T*. *thermophila* MT genes is coordinated in response to a stress situation.

*MTT5* is essential for *T*. *thermophila* viability, in contrast with all MT genes described in a variety of other organisms that appear to be non-essential. For example, *Caenorhabditis elegans* nematodes with knockouts of either of the two MT genes, as well as with both genes disrupted, are still viable [45]. Mice with MT knockouts show normal development and reproduction, but are more vulnerable to oxidative stress [15, 46]. Similarly, knockout strains for all four *Drosophila melanogaster* MT genes are viable but are hypersensitive to Cu^2+^, Cd^2+^ or Zn^2+^ [47].

In *T*. *thermophila*, MTT5 may also have a key role in Pb^2+^ detoxification and adaptation processes. The Pb-affinity of MTT5 has not been determined, but the induction of *MTT5* at 24h Pb^2+^ in the majority of strains is higher than that of other MT isoforms. In addition, basal *MTT5* expression in the Pb-adap strain is increased about 7-fold relative to the control. After 6 months culturing without Pb^2+^, re-exposure for 24h to the Pb^2+^ MTC resulted in higher fold-induction of *MTT5* than of the other MTs.

### The MTT3 isoform: A still little defined MT

*MTT3* is located adjacent to *MTT1* (at 1.7 Kb) on the same macronuclear chromosome and with the same orientation. The two genes (486 bp in length, 85% nucleotide sequence identity) encode similar polypeptides (76% amino acid identity) and they probably originated by paralogous duplication [5, 20, 26]. The sequence changes may have resulted in drastic changes in their metal binding abilities and functions. Moreover, variations in their promoter regions and in their *cis*-regulatory motifs correlate with their differential expression, which may have been an early step in their functional divergence [48]. One difference between their promoter regions is the different number of putative binding sites for AP-1 transcription factors: the *MTT1* promoter contains 6 putative binding sites while the *MTT3* promoter has only 2 [20]. This difference may contribute to the lower gene expression levels of *MTT3* with respect to *MTT1* or *MTT5*, which has 13 putative AP-1 binding sites [6, 26].

*MTT3* is classified as a CdMT because its pattern of Cys residues is very similar to that of other *Tetrahymena* CdMTs [5, 6, 26]. Its expression is mainly induced by Cd^2+^ (after 24h treatments) in almost all strains. However, it is also strongly induced by Cu^2+^ (after 1 or 24h treatment) in the metal-adapted strains, as well as in the Cu-adap (-1M) and (-6M) strains re-exposed to the Cu^2^ MTC ^+^ for 1h. *MTT3* basal expression is quite low, ranked 2^nd^ or 3^rd^ depending on the strain. In the MTT5KD, basal *MTT3* expression was lower than that of the other strains (e.g., 42-fold lower than the control). Although MTT3 has previously been classified as a CdMT and part of the *Tetrahymena* 7a subfamily [6, 20], we would now argue that it may be specialized for Cd^2+^ or Cu^2+^, and may not in fact be particularly well-suited for the coordination of Cd^2+^ [27]. Therefore, the specificity of this MT still remains to be fully defined [27]. The lack of a strong metal preference may provide plasticity that allows MTT3 to perform diverse physiological roles, binding Cd^2+^, Cu^2+^ and/or Zn^2+^ depending on the environment [27].

Unlike any of the other *T*. *thermophila* CdMTs, the MTT3 protein sequence includes 2 histidine residues, while a single histidine is found in a MT from *T*. *patula* [6]. Histidines enhance, through their imidazole rings, the affinity for Zn^2+^ in comparison with Cd^2+^. Histidines are the most frequent Zn^2+^-liganded residues in metalloenzymes [49] and they stabilize the formation of metal-protein complexes in MT proteins [50]. As previously reported, *MTT3* is most strongly induced after Zn^2+^ treatment (1h) [20]. Therefore, this gene may play a role in intracellular homeostasis of essential metals, Zn^2+^ and/or Cu^2+^.

### The MTT2/4 isoforms: Two better than one

*MTT2* and *MTT4*, which are tandemly clustered in the right arm of the micronuclear chromosome 4, share 98% identity at the nucleotide level and 99% at the amino acid level. Their promoter regions are also quite similar (76% identity) and each bears two putative binding motifs for AP-1 transcription factors [6]. Following the duplication that gave rise to the gene pair, there appears to have been little diversification. We do not yet know if the genes are differentially expressed or if both copies are jointly expressed, an increase in gene dosage that could enhance the cellular stress response to metals. Neither seems to be a pseudogene, and the *MTT2* upstream region functions as a copper-inducible promoter [51].

MTT2 and MTT4 isoforms are clearly CuMTs as they show highest affinity to copper (MTT2 > MTT4) among all *T*. *thermophila* MT isoforms, and moreover do not form stable complexes with Cd^2+^ [27]. In almost all the *T*. *thermophila* strains, *MTT2/4* expression is preferentially induced by Cu^2+^, mainly after 1h exposure. The consensus ranking for induction following Cu^2+^ stress is *MTT2/4* > *MTT5* ≈ *MTT3* > *MTT1*. An exception is found in the Cu-adap strain, in which *MTT2/4* genes are ranked last in induction (Fig 7). This is explained by the very high basal expression of *MTT2/4* in that culture compared to the other MT genes: *MTT2/4* basal expression is 4 times higher than *MTT1*, ≈ 16x *MTT3* and ≈ 27x *MTT5*. This elevated *MTT2/4* basal expression may obviate the need to further induce *MTT2/4* upon Cu^2+^ stress. Our results suggest that *MTT2/4* may play a key role in Cu-adaptation.

*MTT2/4* basal expression considerably increases in the KO and/or KD strains. In MTT1KO, *MTT2/4* basal expression levels are increased ≈ 8-fold. In MTT1KO+MTT5KD, the basal expression is increased about 10-fold compared to the control, or 5-fold compared to MTT5KD. These results support the idea of coordinated regulation of MT genes that may include *MTT1*, *MTT5* and *MTT2/4*. In this way, higher *MTT1* basal expression in GFPMTT1 (≈ 2x) may be linked to higher *MTT2/4* basal expression (≈ 7x). Likewise, in GFPMTT5, increased *MTT5* basal expression (≈ 49x) may upregulate basal expression of MTT1 (≈ 5x) and MTT2/4 (≈ 6x).

Overall, our results obtained with *T*. *thermophila* strains and particularly with metal-adapted strains clearly indicate differential roles for the *T*. *thermophila* MT isoforms. Similarly, the four MT isoforms in *Drosophila melanogaster* do not contribute equally to metal detoxification [52]. Most simply, MT functional differentiation can be due to both differential gene expression and to differences in metal binding specificities. We found evidence for inter-connected transcriptional co-regulation of the *T*. *thermophila* MT genes, particularly *MTT1*, *MTT5* and *MTT2/4*. One possibility is that the genes share *trans*-acting regulatory factors, which may be directly or indirectly controlled by the MTs themselves, leading to activation or repression of MT gene expression. In recent years, the traditional view that MTs are exclusively involved in metal detoxification is being replaced by the idea that they are dynamically involved in a range of phenomena including gene regulation, neurotransmission, control of neurodegenerative and neoplastic disorders, and tumor progression [53]. These functions may depend upon the roles of MTs in pathways including intracellular transport, signaling, essential metal homeostasis, enzymatic and transcriptional regulation, as well as metal detoxification [53]. These roles may depend on the formation of protein complexes that include MTs. Mammalian MTs have been reported to directly interact with proteins including Rab3A GTPase [54], LPR-receptors [55], bovine serum albumin [56] and p53 and NF-kB transcription factors [57]. Moreover, MTs can also interact indirectly with other proteins, swapping essential ions (Zn, Cu or Fe) with proteins including ferritin [58], Zn-dependent enzymes [59] and Zn-finger transcription factors [60, 61].

Finally, it is important to note that MTs are not uniquely responsible for metal detoxification. Other cellular mechanisms are involved in the stress response, such as active transport by metal efflux-pumps. Interestingly, the *T*. *thermophila* genome includes 485 genes that encode putative membrane transporters for inorganic cations [62], and some may have important functions in metal detoxification.

## Conclusions

1- After carrying out a comparative MT gene expression analysis using different *T*. *thermophila* strains, we can distinguish differential roles among the five MT isoforms of this ciliate. a)- MTT1 protein is the *T*. *thermophila* MT with the highest affinity for Cd^2+^, and *MTT1* is primarily induced by Cd^2+^. Under no-stress conditions, *MTT1* shows the highest basal expression in all strains except for the Cu-adap. It can be considered as a MT gene probably involved in metal cell homeostasis, but also with an important detoxification role, because it is ranked second or third in the consensus pattern of MT gene expression induction under Cd^2+^ or Pb^2+^ stress. This gene is not essential, but cells lacking *MTT1* show higher sensitivity to Cd^2+^. b)- *MTT5* basal expression is the lowest in several of the *T*. *thermophila* strains. However, under metal stress, it is the MT gene with the highest induction. When *MTT5* is induced, the rest of the MT gene isoforms are over-expressed as well, suggesting that there may be coordination between the *MTT5* product and induction of other MT genes under metal stress. *MTT5* might be considered as an "alarm” MT gene, that is over-expressed under metal stress (mainly Cd^2+^ or Pb^2+^) and promotes the expression of other MT genes. *MTT5* is essential because it was not possible to isolate a stable knockout strain. Therefore, this is the first time that a MT gene appears to be essential. c)- *MTT3* is preferably induced by Cd^2+^ and Cu^2+^ and the protein has an ambiguous affinity for these two metals. We consider it to be an "undefined" MT [27] and the lack of a specific metal preference may maintain plasticity, allowing it to develop diverse physiological roles, binding Cd^2+^, Cu^2+^ or Zn^2+^ depending on the environmental conditions. In addition, a possible role for this MT isoform could be the intracellular homeostasis of essential metals. d)- *MTT2* and *MTT4* are almost identical and they encode CuMTs with high affinity to Cu^2+^. Moreover, these genes are over-expressed under Cu^2+^ treatments in almost all strains. A significant increase in basal *MTT2/4* expression is detected in the Cu-adap, as well as when some other MT genes have been disrupted or knocked down. The maintenance of two nearly identical genes may represent an adaptation to increase the total levels of cytoplasmic CuMTs.

2- *T*. *thermophila* MT genes (primarily *MTT1*, *MTT5* and *MTT2/4*) may be connected in a transcriptional regulatory network. The details of this putative network are unknown but may involve interactions between AP-1 transcription factors, metal ions and MT proteins.

3- Cell adaptation to Cd^2+^ leads to up-regulation of *MTT1*, while Cu^2+^ or Pb^2+^ adaptation involves up-regulation of *MTT2/4* or *MTT5* expression, respectively. This is consistent with the three genes having specific roles in adaptation to different metals.

## Supporting information

S1 Fig Ranking of basal expression levels under no-metal conditions for the five *T*. *thermophila* MT genes. The predominant consensus pattern is shown.
(TIF)

S1 Table Primers used in this study. ^(^*^)^: MTT2QA and MTT2QB primers were used to amplify *MTT2* and *MTT4* CuMT genes indistinctly because both have very similar nucleotide sequences (98% identity) and it was not possible to design specific primers for each of them.
(DOCX)

S2 Table Quantitative RT-PCR standard-curve parameters. (*): correlation coefficient. Efficiency (E) is calculated from the slope value of the standard curve: E = 10^(-1/slope)-1^.
(DOCX)

S3 Table MT gene induction values obtained by qRT-PCR after different metal treatments. (-): Data not obtained. Normalization of the gene expression was carried out using the β-actin as an endogenous control gene. We show the average value ± standard deviation of two or three independent experiments. (-1M) or (-6M): metal adapted strains after 1 or 6 months in growth medium without metal exposure.
(DOCX)

S4 Table Comparison of the C_t_ values obtained in control situations (no metal exposure) from the different *T*. *thermophila* strains analyzed in the comparative MT gene expression study. *β-actin* gene was used as an endogenous control and it was considered a reference for neutralizing the variability of the qPCR technique. ^(^*^)^ C_t_ values that are considerably lower than those obtained in the control SB1969 strain. (-): not applicable, because the MTT1KO and the MTT1KO + MTT5KD strains have lost all the copies of the *MTT1* gene. (-1M) or (-6M): these parameters were calculated after maintaining metal adapted strains 1 or 6 months in growth medium without metal exposure.
(DOCX)

S5 Table Comparison of the basal expression levels among different MT gene isoforms in each *T*. *thermophila* strain. Differences among basal expression levels for the different MT genes in each *T*. *thermophila* strain were calculated using the following formula: 2^(Ct1-Ct2)^, being C_t1_ and C_t2_ the C_t_ values under a control situation (no metal exposure) between two MT genes in the same strain. We compared in each strain all MT gene basal expression levels by twos, distinguishing them by two colours: red and green. For each comparison, results are indicated in red or green depending on the MT gene that has a higher basal expression level in the same strain. Comparison values higher than 4 (C_t_ value differences higher than 2 cycles) are shaded in grey. (-): not applicable. (-1M) or (-6M): these parameters were calculated after maintaining metal adapted strains 1 or 6 months in growth medium without metal exposure.
(DOCX)

S6 Table Comparison of the basal expression levels of each MT gene among different *T*. *thermophila* strains. Differences among basal expression levels for each MT gene among different *T*. *thermophila* strains were calculated using the following formula: 2^(Ct1-Ct2)^, being C_t1_ and C_t2_ the C_t_ values under a control situation (no metal exposure) for the same MT gene into two different strains. We compared all the *T*. *thermophila* analyzed strains by twos, distinguishing them by two colours: red and green. For each comparison, results are indicated in red or green depending on the strain that has a higher basal expression level for the same MT gene. Comparison values which are higher than 4 (C_t_ value differences higher than 2 cycles) are shaded in grey. (-): not applicable. (-1M) or (-6M): these parameters were calculated after maintaining metal adapted strains 1 or 6 months in growth medium without metal exposure.
(DOCX)

## References

[1] Gadd GM. Heavy metal pollutants: environmental and biotechnological aspects In: Lederberg J., editor. Encyclopedia of Microbiology. New Yok: Academic Press 2000 pp. 607–617.

[2] Leonard SS, Harris GK, Shi X. Metal-induced oxidative stress and signal transduction. Free Radic Biol Med. 2004;12: 1921–1942. 10.1016/j.freeradbiomed.2004.09.010 15544913

[3] Valko M, Morris H, Cronin MT. Metals, toxicity and oxidative stress. Curr Med Chem. 2005;12: 1161–1208. 15892631 10.2174/0929867053764635

[4] Wysocki R, Tamás MJ. How *Saccharomyces cerevisiae* copes with toxic metals and metalloids. FEMS Microbiol Rev. 2010;34: 925–951. doi: 10.1111/j.1574-6976.2010.00217.x 20374295 10.1111/j.1574-6976.2010.00217.x

[5] Gutiérrez JC, Amaro F, Díaz S, de Francisco P, Cubas LL, Martín-González A. Ciliate metallothioneins: unique microbial eukaryotic heavy-metal-binder molecules. J Biol Inorg Chem. 2011;16: 1025–1034. doi: 10.1007/s00775-011-0820-9 21785894 10.1007/s00775-011-0820-9

[6] De Francisco P, Melgar LM, Díaz S, Martín-González A, Gutiérrez JC. The *Tetrahymena* metallothionein gene family: twenty-one new cDNAs, molecular characterization, phylogenetic study and comparative analysis of the gene expression under different abiotic stressors. BMC Genomics. 2016;17: 1–23. doi: 10.1186/s12864-015-2294-6 27165301 10.1186/s12864-016-2658-6 PMC4862169

[7] Sakulsak N. Metallothionein: An overview on its metal homeostatic regulation in mammals. Int J Morphol. 2012;30: 1007–1012.

[8] Liu J, Klaassen CD. Absorption and distribution of cadmium in metallothionein-I transgenic mice. Fund Appl Toxicol. 1969;29: 294–300. 10.1006/faat.1996.0034 8742328

[9] Viarengo A, Burlando B, Ceratto N, Panfoli I. Antioxidant role of metallothioneins: a comparative overview. Cell Mol Biol. 2000;46: 407–417. 10774929

[10] Miles AT, Hawksworth GM, Beattie JH, Rodilla V. Induction, regulation, degradation, and biological significance of mammalian metallothioneins. Crit Rev Biochem Mol Biol. 2000;35: 35–70. doi: 10.1080/10409230091169168 10755665 10.1080/10409230091169168

[11] Penkowa M, Carrasco J, Giralt M, Molinero A, Hernández J, Campbell IL, et al Altered central nervous system cytokine-growth factor expression profiles and angiogenesis in metallothionein-I + II deficient mice. J Cereb Blood Flow Metab. 2000;20: 1174–1189. doi: 10.1097/00004647-200008000-00003 10950378 10.1097/00004647-200008000-00003

[12] Vidal F, Hidalgo J. Effect of zinc and copper on preimplantation mouse embryo development in vitro and metallothionein levels. Zygote. 1993;1: 225–229. 8081819 10.1017/s0967199400001507

[13] Coyle P, Philcox JC, Carey LC and Rofe AM. Metallothionein: the multipurpose protein. Cell Mol Life Sci. 2002;59: 627–647. 12022471 10.1007/s00018-002-8454-2 PMC11337511

[14] Huggins CJ, Mayekar MK, Martin N, Saylor KL, Gonit M, Jailwala P, et al C/EBPγ is a critical regulator of cellular stress response networks through heterodimerization with ATF4. Mol Cell Biol. 2016;36: 693–713. 10.1128/MCB.00911-15 PMC4760225 26667036

[15] Klaassen CD, Liu J. Induction of metallothionein as an adaptive mechanism affecting the magnitude and progression of toxicological injury. Environ Health Perspect. 1998;106: 297–300. 9539022 10.1289/ehp.98106s1297 PMC1533300

[16] Pedrini-Martha V, Schenegg R, Baurand PE, deVaufleury A, Dallinger R. The physiological role and toxicological significance of the non-metal-selective cadmium/copper-metallothionein isoform differ between embryonic and adult helicid snails. Comp Biochem Phisiol C. 2017;199: 38–47. 10.1016/j.cbpc.2017.02.009 28254493

[17] Amaro F, de Lucas MP, Martín-González A, Gutiérrez JC. Two new members of the *Tetrahymena* multi-stress-inducible metallothionein family: Characterization and expression analysis of *T*. *rostrata* Cd/Cu metallothionein genes. Gene. 2008;423: 85–91. doi: 10.1016/j.gene.2008.07.002 18675326 10.1016/j.gene.2008.07.002

[18] Boldrin F, Santovito G, Irato P, Piccinni E. Metal interaction and regulation of *Tetrahymena pigmentosa* metallothionein genes. Protist. 2002;153: 283–291. doi: 10.1078/1434-4610-00105 12389817 10.1078/1434-4610-00105

[19] Cheung A, Pok L, Vincent KLL, King MC. Tilapia metallothionein genes: PCR-cloning and gene expression studies. Biochim Biophys Acta. 2005;1731: 191–201. doi: 10.1016/j.bbaexp.2005.09.006 16309756 10.1016/j.bbaexp.2005.09.006

[20] Díaz S, Amaro F, Rico D, Campos V, Benitez L, Martín-González A, et al *Tetrahymena* metallothioneins fall into two discrete subfamilies. PloS One. 2007;2: e291 doi: 10.1371/journal.pone.0000291 17356700 10.1371/journal.pone.0000291 PMC1808422

[21] Dondero F, Cavaletto M, Chezzi AR, La Terza A, Banni M, Viarengo A. Biochemical characterization and quantitative gene expression analysis of the multi-stress inducible metallothionein from *Tetrahymena thermophila*. Protist. 2004;155: 157–168. doi: 10.1078/143446104774199565 15305793 10.1078/143446104774199565

[22] Fu C, Miao W. Cloning and characterization of a new multi-stress inducible metallothionein gene in *Tetrahymena pyriformis*. Protist. 2006;157: 193–203. doi: 10.1016/j.protis.2006.02.006 16621695 10.1016/j.protis.2006.02.006

[23] Guo L, Fu C, Miao W. Cloning, characterization, and gene expression analysis of a novel cadmium metallothionein gene in *Tetrahymena pigmentosa*. Gene. 2008;423: 29–35. doi: 10.1016/j.gene.2008.04.023 18675523 10.1016/j.gene.2008.04.023

[24] Hughes S, Sturzenbaum SR. Single and double metallothionein knockout in the nematode *C*. *elegans* reveals cadmium dependent and independent toxic effects on life history traits. Environ Pollut. 2007;145: 395–400. doi: 10.1016/j.envpol.2006.06.003 17141712 10.1016/j.envpol.2006.06.003

[25] Masters BA, Kelly EJ, Quaife CJ, Brinster RL, Palmiter RD. Targeted disruption of metallothionein I and II genes increases sensitivity to cadmium. Proc Natl Acad Sci USA. 1994;91: 548–588. 10.1073/pnas.91.2.584 PMC42993 8290567

[26] Gutiérrez JC, Amaro F, Martín-González A. From heavy metal-binders to biosensors: Ciliate metallothioneins discussed. BioEssays. 2009;31: 805–816. doi: 10.1002/bies.200900011 19492353 10.1002/bies.200900011

[27] Espart A, Marín M, Gil-Moreno S, Palacios O, Amaro F, Martín-González A, et al Hints for metal-preference protein sequence determinants: different metal binding features of the five *Tetrahymena thermophila* metallothioneins. Int J Biol Sci. 2015;11: 456–471. doi: 10.7150/ijbs.11060 25798065 10.7150/ijbs.11060 PMC4366644

[28] Adamo GM, Brocca S, Passolunghi S, Salvato B, Lotti M. Laboratory evolution of copper tolerant yeast strains. Microb Cell Fact. 2012;11: 1–11. doi: 10.1186/1475-2859-11-1 22214286 10.1186/1475-2859-11-1 PMC3276424

[29] Butler PR, Brown M, Oliver SG. Improvement of antibiotic titers from *Streptomyces* bacteria by interactive continuous selection. Biotechnol Bioeng. 1996;49: 185–196. doi: 10.1002/(SICI)1097-0290(19960120)49:2<185::AID-BIT7>3.0.CO;2-M 18623568 10.1002/(SICI)1097-0290(19960120)49:2<185::AID-BIT7>3.0.CO;2-M

[30] Kumar G, Kuswaha HR, Panjabi-Sabharwal V, Kumari S, Joshi R, Karan R, et al Clustered metallothionein genes are co-regulated in rice and ectopic expression of *OsMT1e-P* confers multiple abiotic stress tolerance in tobacco via ROS scavenging. BMC Plant Biology. 2012; 12: 107 doi: 10.1186/1471-2229-12-107 22780875 10.1186/1471-2229-12-107 PMC3491035

[31] Costa D, Mariën J, Janssens TKS, van Gestel CAM, Driessen G, Sousa JP, et al Influence of adaptive evolution of cadmium tolerance on neutral and functional genetic variation in *Orchesella cincta*. Ecotoxicology. 2012;21: 2078–2087. doi: 10.1007/s10646-012-0961-9 22717685 10.1007/s10646-012-0961-9

[32] Minty JJ, Lesnefsky AA, Lin F, Chen Y, Zaroff TA, Veloso AB, et al Evolution combined with genomic study elucidates genetic bases of isobutanol tolerance in *Escherichia coli*. Microb Cell Fact. 2011; 10: 18 doi: 10.1186/1475-2859-10-18 21435272 10.1186/1475-2859-10-18 PMC3071312

[33] Van Maris AJ, Winkler AA, Kuyper M, de Laat WT, van Dijken JP, Pronk JT. Development of efficient xylose fermentation in *Saccharomyces cerevisiae*: xylose isomerase as a key component. Adv Biochem Eng Biotechnol. 2007;108: 179–204. doi: 10.1007/10_2007_057 17846724 10.1007/10_2007_057

[34] Guimaraes PM, Francois J, Parrou JL, Teixeira JA, Domingues L. Adaptive evolution of a lactose-consuming *Saccharomyces cerevisiae* recombinant. Appl Environ Microbiol. 2008;74: 1748–1756. doi: 10.1128/AEM.00186-08 18245248 10.1128/AEM.00186-08 PMC2268319

[35] Capecchi MR. Altering the genome by homologous recombination. Science. 1989;244: 1288–1292. 2660260 10.1126/science.2660260

[36] Amaro F, Turkewitz AP, Martín-González A., Gutiérrez JC. Functional GFP-metallothionein fusion protein from *Tetrahymena thermophila*: a potential whole-cell biosensor for monitoring heavy metal pollution and a cell model to study metallothionein overproduction effects. Biometals. 2014;27: 195–205. doi: 10.1007/s10534-014-9704-0 24430977 10.1007/s10534-014-9704-0 PMC4707044

[37] Kataoka K, Schoeberl UE and Mochizuki K. Modules for C-terminal epitope tagging of *Tetrahymena* genes. J Microbiol Methods. 2010;82: 342–346. doi: 10.1016/j.mimet.2010.07.009 20624430 10.1016/j.mimet.2010.07.009 PMC2935961

[38] Cassidy-Hanley D, Bowen J, Lee JH, Cole E, VerPlank LA, Gaertig J, et al Germline and somatic transformation of mating *Tetrahymena thermophila* by particle bombardment. Genetics. 1997;146: 135–147. 9136007 10.1093/genetics/146.1.135 PMC1207932

[39] Larionov A, Krause A, Miller W. A standard curve based method for relative real time PCR data processing. BMC Bioinform. 2005;6: 62. 10.1186/1471-2105-6-62 PMC1274258 15780134

[40] Huang Y, Agrawal AF. Experimental evolution of gene expression and plasticity in alternative selective regimes. PloS Genet. 2016;12: 1–23. 10.1371/journal.pgen.1006336 PMC5035091 27661078

[41] Timmermans MJ, Ellers J, Roelofs D, van Straalen NM. Metallothionein mRNA expression and cadmium tolerance in metal-stressed and reference populations of the springtail *Orchesella cincta*. Ecotoxicology. 2005;14: 727–739. doi: 10.1007/s10646-005-0020-x 16160751 10.1007/s10646-005-0020-x

[42] Liu N, Scott JG. Increased transcription of CYP6D1 causes cytochrome P450-mediated insecticide resistance in house fly. Insect Biochem Mol Biol. 1998;28: 531–535. 9753764 10.1016/s0965-1748(98)00039-3

[43] Niederwanger M, Dvorak M, Schnegg R, Pedrini-Martha V, Bacher K, Bidoli M, Dallinger R. Challenging the metallothionein (MT) gene of *Biomphalaria glabrata*: unexpected response patterns due to cadmium exposure and temperature stress. J Mol Sci. 2017;18: 1747. 10.3390/ijms18081747 PMC5578137 28800079

[44] Van Straalen NM, Janssens TKS, Roelofs D. Micro-evolution of toxicant tolerance: from single genes to the genome's tangled bank. Ecotoxicology. 2011;20: 574–579. doi: 10.1007/s10646-011-0631-3 21416112 10.1007/s10646-011-0631-3 PMC3081431

[45] Höckner M, Dallinger R, Stürzenbaum SR. Nematode and snail metallothioneins. J Biol Inorg Chem. 2011;16: 1057–1065. doi: 10.1007/s00775-011-0826-3 21822727 10.1007/s00775-011-0826-3

[46] Michalska AE, Choo KHA. Targeting and germ-line transmission of a null mutation at the metallothionein I and II loci in mouse. Proc Natl Acad Sci USA. 1993;90: 8088–8092. 8367468 10.1073/pnas.90.17.8088 PMC47293

[47] Egli D, Yepiskoposyan H, Selvaraj A, Balamurugan K, Rajaram R, Simons A, et al A family knockout of all four *Drosophila* metallothioneinas reveals a central role in copper homeostasis and detoxification. Mol Cell Biol. 2006;26: 2286–2296. doi: 10.1128/MCB.26.6.2286-2296.2006 16508004 10.1128/MCB.26.6.2286-2296.2006 PMC1430275

[48] Li WH, Yang J, Gu X. Expression divergence between duplicated genes. Trends Genet. 2005;21: 602–607. doi: 10.1016/j.tig.2005.08.006 16140417 10.1016/j.tig.2005.08.006

[49] Daniels MJ, Turner-Cavet JS, Selkirk R, Sun H, Parkinson JA, Sadler PJ, et al Coordination of Zn^2+^ (and Cd^2+^) by prokaryotic metallothionein. Involvement of His-Imidazole. J Biol Chem. 1998;273: 22957–22961. 9722517 10.1074/jbc.273.36.22957

[50] Blindauer CA. Metallothioneins with unusual residues: histidines as modulators of zinc affinity and reactivity. J Inorg Biochem. 2008;102: 507–21. doi: 10.1016/j.jinorgbio.2007.10.032 18171588 10.1016/j.jinorgbio.2007.10.032

[51] Boldrin F, Santovito G, Gaertig J, Wloga D, Cassidy-Hanley D, Clark TG, et al Metallothionein gene from *Tetrahymena thermophila* with a copper-inducible-repressible promoter. Eukaryot Cell. 2006;5: 422–425. doi: 10.1128/EC.5.2.422-425.2006 16467482 10.1128/EC.5.2.422-425.2006 PMC1405887

[52] Egli D, Domènech J, Selvaraj A, Balamurugan K, Hua H, Capdevila M, et al The four members of the *Drosophila* metallothionein family exhibit distinct overlapping roles in heavy metal homeostasis and detoxification. Genes Cell. 2006;11: 647–658. 10.1111/j.1365-2443.2006.00971.x 16716195

[53] Atrian S, Capdevila M. Metallothionein-protein interactions. BioMol Concepts. 2013;4: 143–160. doi: 10.1515/bmc-2012-0049 25436572 10.1515/bmc-2012-0049

[54] Knipp M, Meloni G, Roschitzki B, Vasak M. Zn_7_-metallothionein-3 and the synaptic vesicle cycle: interaction of metallothionein-3 with the small GTPase Rab3A. Biochemistry. 2005;44: 3159–3165. doi: 10.1021/bi047636d 15736926 10.1021/bi047636d

[55] Ambjørn M, Asmussen JW, Lindstam M, Gotfryd K, Jacobsen C, Kiselyov VV, et al Metallothionein and a peptide modeled after metallothionein, EmtinB, induce neuronal differentiation and survival through binding to receptors of the low-density lipoprotein receptor family. J Neurochem. 2008;104: 21–37. doi: 10.1111/j.1471-4159.2007.05036.x 17986228 10.1111/j.1471-4159.2007.05036.x

[56] Quiming NS, Vergel RB, Nicolas MG, Villanueva JA. Interaction of bovine serum albumin and metallothionein. J Health Sci. 2005;51: 8–15.

[57] Ostrakhovitch EA, Olsson PE, Jiang S, Cherian MG. Interaction of metallothionein with tumor suppressor p53 protein. FEBS Lett. 2006;580: 1235–1238. doi: 10.1016/j.febslet.2006.01.036 16442532 10.1016/j.febslet.2006.01.036

[58] Orihuela R, Fernández B, Palacios O, Valero E, Atrian S, Watt RK, et al Ferritin and metallothionein: dangerous liaisons. Chem Commun. 2011;47: 12155–12157. 10.1039/c1cc14819b 21991581

[59] Maret W, Jacob C, Vallee BL, Fisher EH. Inhibitory sites in enzymes: zinc removal and reactivation by thionein. Proc Natl Acad Sci USA. 1999;96: 1936–1940. 10051573 10.1073/pnas.96.5.1936 PMC26715

[60] Huang M, Shaw IF, Petering DH. Interprotein metal exchange between transcription factor IIIa and apo-metallothionein. J Inorg Biochem. 2004;98: 639–648. doi: 10.1016/j.jinorgbio.2004.02.004 15041244 10.1016/j.jinorgbio.2004.02.004 PMC3535305

[61] Zeng J, Vallee BL, Kägi JHR. Zinc transfer from transcription factor IIIA fingers to thionein clusters. Proc Natl Acad Sci USA. 1991;88: 9984–9988. 1835092 10.1073/pnas.88.22.9984 PMC52851

[62] Eisen JA, Coyne RS, Wu M, Wu D, Thiagarajan M, Wortman JR, et al Macronuclear genome sequence of the ciliate *Tetrahymena thermophila*, a model eukaryote. PLoS Biol, 2006;4: c286. 10.1371/journal.pbio.0040286 PMC1557398 16933976

