# Supplementary figures and images for: Extreme metal adapted, knockout and knockdown strains reveal a coordinated gene expression among different Tetrahymena thermophila metallothionein isoforms
Source: PLoS One. 2017 Dec 5;12(12):e0189076. doi: 10.1371/journal.pone.0189076 (PMC5716537; doi:10.1371/journal.pone.0189076)

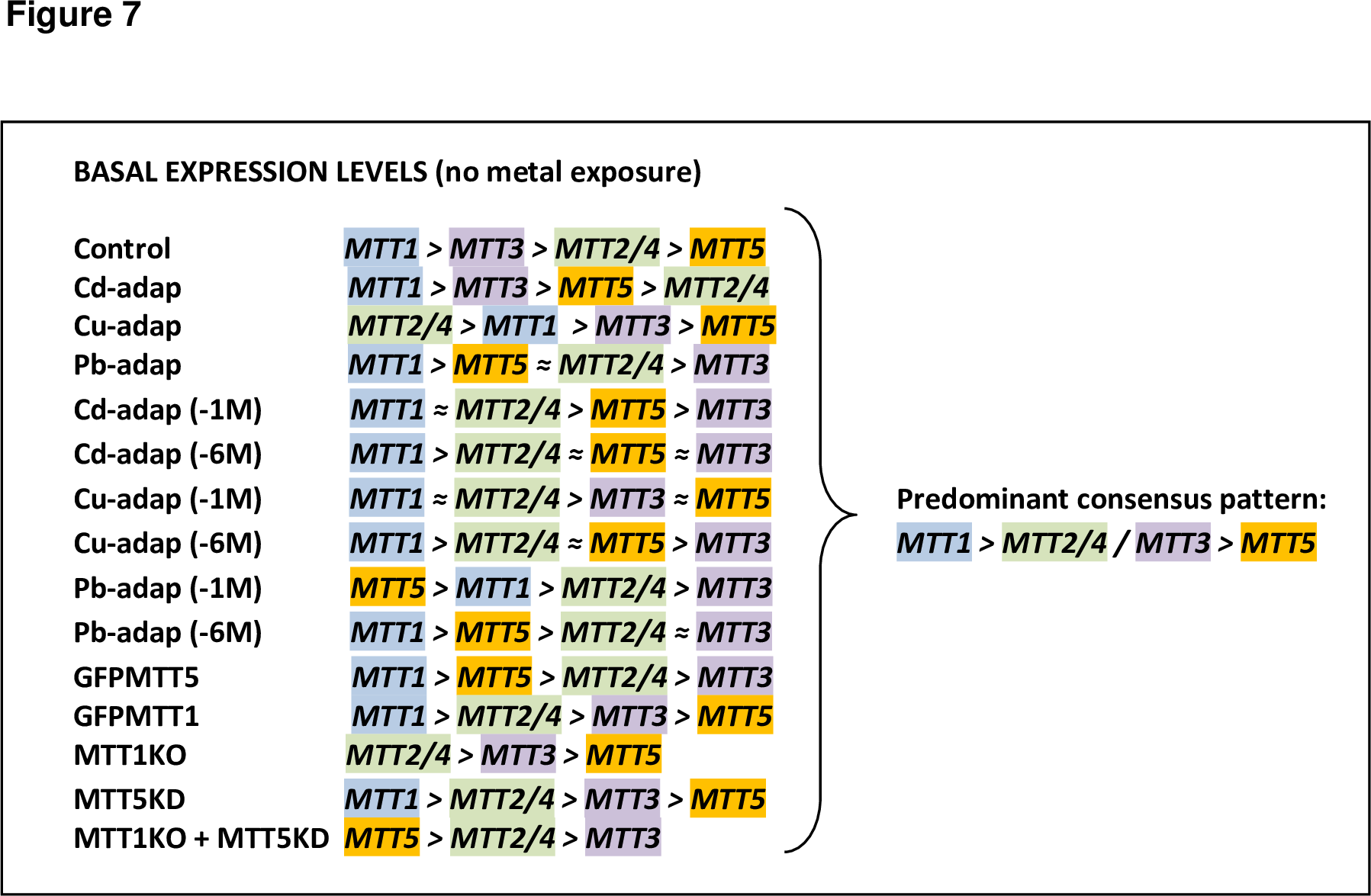

Supplement: S1 Fig — The predominant consensus pattern is shown. (TIF) [file pone.0189076.s001.tif]
