# Supplementary material for: Extreme metal adapted, knockout and knockdown strains reveal a coordinated gene expression among different Tetrahymena thermophila metallothionein isoforms
Source: PLoS One. 2017 Dec 5;12(12):e0189076. doi: 10.1371/journal.pone.0189076 (PMC5716537; doi:10.1371/journal.pone.0189076)
Supplement: S1 Table — (*): MTT2QA and MTT2QB primers were used to amplify MTT2 and MTT4 CuMT genes indistinctly because both have very similar nucleotide sequences (98% identity) and it was not possible to design specific primers for each of them. (DOCX) [file pone.0189076.s002.docx]

**S1 Table.** Primers used in this study

| **Primer** | **Sequence (5´- 3´)** | **Use** |
| --- | --- | --- |
| 5UTRMTT1A  5UTRMTT1B | GAAAAAAGCGGCCGCTTAGTGCACAATGTTTGAATGT  AAAACTGCAGGGATCATTGCATAAATCGTGAAT | PCR 5'UTR of *MTT1* gene  (construct for the MTT1KO) |
| 3UTRMTT1A  3UTRMTT1B | CCCAAGCTTCATAATTGCATAAAATCAGTTTCG  CCGCTCGAGTCCAAAGATGTTTATGAGATAAATACA | PCR 3'UTR of *MTT1* gene  (construct for the MTT1KO) |
| 5UTRMTT5A  5UTRMTT5B | GAAAAAAGCGGCCGCGTTGTAAATTTACAAATTCTAAT  AAAACTGCAGTGGTGAACTCATGATTGTATTCAAG | PCR 5'UTR of *MTT5* gene  (constructs for the MTT5KD and the MTT1KO+MTT5KD) |
| 3UTRMTT5A  3UTRMTT5B | CCCAAGCTTTGCAATCAAATGAATGATTAA  CCGCTCGAGTTGAAATTCCTGATGATGGTTT | PCR 3'UTR of *MTT5* gene  (constructs for the MTT5KD and the MTT1KO+MTT5KD) |
| TtACT1  TtACT2 | CTCTCTTTCTACCTTCCAAACT  AGGACCAGATTCATCATATTC | qRT-PCR of the β-actin gene |
| MTT1QA  MTT1QB | ATGGATAAAGTTAATAGCTGTTGCTG  AAAGCAGCAGGGTTTAG | qRT-PCR of the *MTT1* gene |
| MTT3QA  MTT3QB | ACAATGTAATTGTGCTT  TAAGCAGCTGGATTTGA | qRT-PCR of the *MTT3* gene |
| MTT5QA  MTT5QB | TGTGTCGGTTCAGGAGAAGGA  AGGTCCGCATTTACATTCAGCTT | qRT-PCR of the *MTT5* gene |
| MTT2QA*  MTT2QB* | ATGCAAATGTGGATCTC  CAGTTGGAAGTAGAACC | qRT-PCR of the *MTT2/MTT4* genes* |

^(^*^)^: MTT2QA and MTT2QB primers were used to amplify *MTT2* and *MTT4* CuMT genes indistinctly because both have very similar nucleotide sequences (98% identity) and it was not possible to design specific primers for each of them.
