# Supplementary material for: Extreme metal adapted, knockout and knockdown strains reveal a coordinated gene expression among different Tetrahymena thermophila metallothionein isoforms
Source: PLoS One. 2017 Dec 5;12(12):e0189076. doi: 10.1371/journal.pone.0189076 (PMC5716537; doi:10.1371/journal.pone.0189076)
Supplement: S2 Table — (*): correlation coefficient. Efficiency (E) is calculated from the slope value of the standard curve: E = 10(-1/slope)-1. (DOCX) [file pone.0189076.s003.docx]

**S2 Table.** Quantitative RT-PCR standard-curve parameters

| **Strain** | **Gene** | **Slope** | **Efficiency (%)** | **Y-intercept** | **R^2^ ^(*)^** |
| --- | --- | --- | --- | --- | --- |
| **Control**  **SB1969** | *Β-actin* | -3.154 | 107.5 | 11.292 | 0.983 |
|  | *MTT1* | -3.160 | 107.2 | 6.351 | 0.994 |
|  | *MTT2/4* | -3.476 | 94.0 | 10.449 | 0.997 |
|  | *MTT3* | -3.506 | 92.9 | 10.022 | 0.992 |
|  | *MTT5* | -3.602 | 89.5 | 7.897 | 0.955 |
| **GFPMTT1** | *Β-actin* | -3.436 | 95.4 | 11.311 | 0.998 |
|  | *MTT1* | -3.495 | 93.3 | 6.742 | 0.992 |
|  | *MTT2/4* | -3.451 | 94.9 | 10.46 | 0.997 |
|  | *MTT3* | -3.357 | 98.6 | 9.678 | 0.994 |
|  | *MTT5* | -3.546 | 91.4 | 9.098 | 0.997 |
| **GFPMTT5** | *ACTIN* | -3.541 | 91.6 | 10.006 | 0.987 |
|  | *MTT1* | -3.428 | 95.8 | 6.218 | 0.992 |
|  | *MTT2/4* | -3.539 | 91.7 | 10.513 | 0.991 |
|  | *MTT3* | -3.510 | 92.7 | 8.198 | 0.997 |
|  | *MTT5* | -3.479 | 93.8 | 5.507 | 0.985 |
| **Cd-adap** | *Β-actin* | -3.393 | 97.1 | 15.985 | 0.999 |
|  | *MTT1* | -3.515 | 92.5 | 12.751 | 0.996 |
|  | *MTT2/4* | -3.560 | 91.0 | 18.425 | 0.997 |
|  | *MTT3* | -3.317 | 100.2 | 7.255 | 0.993 |
|  | *MTT5* | -3.678 | 87.0 | 19.316 | 0.997 |
| **Cu-adap** | *Β-actin* | -3.462 | 94.5 | 15.267 | 0.998 |
|  | *MTT1* | -3.451 | 94.9 | 12.845 | 0.999 |
|  | *MTT2/4* | -3.371 | 98.0 | 7.951 | 0.998 |
|  | *MTT3* | -3.404 | 96.7 | 10.446 | 0.991 |
|  | *MTT5* | -3.415 | 96.2 | 13.371 | 0.995 |
| **Pb-adap** | *Β-actin* | -3.401 | 96.8 | 13.248 | 0.999 |
|  | *MTT1* | -3.539 | 91.7 | 12.760 | 0.999 |
|  | *MTT2/4* | -3.586 | 90.0 | 15.841 | 0.994 |
|  | *MTT3* | -3.596 | 89.7 | 16.711 | 0.996 |
|  | *MTT5* | -3.480 | 93.8 | 9.242 | 0.979 |
| **Cd-adap**  **(1 month without Cd)** | *Β-actin* | -3.520 | 98.8 | 11.142 | 0.966 |
|  | *MTT1* | -3.380 | 97.6 | 14.125 | 0.986 |
|  | *MTT2/4* | -3.467 | 94.3 | 14.561 | 0.976 |
|  | *MTT3* | -3.531 | 92.0 | 8.210 | 0.991 |
|  | *MTT5* | -3.436 | 95.5 | 7.359 | 0.986 |
| **Cu-adap**  **(1 month without Cu)** | *Β-actin* | -3.521 | 92.3 | 14.140 | 0.984 |
|  | *MTT1* | -3.789 | 93.6 | 10.689 | 0.988 |
|  | *MTT2/4* | -3.965 | 79.0 | 8.424 | 0.998 |
|  | *MTT3* | -3.418 | 96.1 | 15.17 | 0.964 |
|  | *MTT5* | -3.341 | 99.2 | 16.793 | 0.945 |
| **Pb-adap**  **(1 month without Pb)** | *Β-actin* | -3.152 | 107.6 | 13.665 | 0.999 |
|  | *MTT1* | -3.310 | 100.5 | 9.956 | 0.998 |
|  | *MTT2/4* | -3.659 | 88.0 | 15.836 | 0.999 |
|  | *MTT3* | -3.895 | 80.6 | 12.576 | 0.987 |
|  | *MTT5* | -3.384 | 97.5 | 7.592 | 0.982 |

| **Strain** | **Gene** | **Slope** | **Efficiency (%)** | **Y-intercept** | **R^2^ ^(*)^** |
| --- | --- | --- | --- | --- | --- |
| **Cd-adap**  **(6 months without Cd)** | *Β-actin* | -3.661 | 87.6 | 11.194 | 0.975 |
|  | *MTT1* | -3.628 | 88.6 | 13.328 | 0.946 |
|  | *MTT2/4* | -3.777 | 84.0 | 16.227 | 0.985 |
|  | *MTT3* | -3.860 | 82.0 | 16.308 | 0.991 |
|  | *MTT5* | -3.988 | 78.1 | 14.955 | 0.956 |
| **Cu-adap**  **(6 months without Cu)** | *Β-actin* | -3.497 | 93.2 | 14.077 | 0.993 |
|  | *MTT1* | -3.610 | 89.2 | 12.082 | 0.986 |
|  | *MTT2/4* | -3.596 | 90.0 | 12.049 | 0.988 |
|  | *MTT3* | -3.399 | 97.0 | 9.519 | 0.997 |
|  | *MTT5* | -3.433 | 95.5 | 10.2 | 0.994 |
| **Pb-adap**  **(6 months without Pb)** | *Β-actin* | -3.350 | 98.8 | 15.775 | 0.948 |
|  | *MTT1* | -3.187 | 105.9 | 19.879 | 0.997 |
|  | *MTT2/4* | -3.675 | 87.0 | 18.302 | 0.975 |
|  | *MTT3* | -3.055 | 113.0 | 24.119 | 0.999 |
|  | *MTT5* | -3.367 | 98.1 | 18.158 | 0.98 |
| **MTT1KO** | *Β-actin* | -3.457 | 94.6 | 14.775 | 0.994 |
|  | *MTT2/4* | -3.428 | 95.7 | 9.453 | 0.995 |
|  | *MTT3* | -3.701 | 86.1 | 9.213 | 0.994 |
|  | *MTT5* | -4.059 | 76.4 | 5.658 | 0.992 |
| **MTT5KD** | *Β-actin* | -3.592 | 89.9 | 10.963 | 0.987 |
|  | *MTT1* | -3.554 | 91.1 | 5.988 | 0.990 |
|  | *MTT2/4* | -3.626 | 88.7 | 10.249 | 0.990 |
|  | *MTT3* | -3.861 | 81.6 | 10.192 | 0.996 |
|  | *MTT5* | -3.765 | 84.3 | 13.024 | 0.993 |
| **MTT1KO + MTT5KD** | *Β-actin* | -3.280 | 102.8 | 11.972 | 0.996 |
|  | *MTT2/4* | -3.632 | 88.5 | 10.093 | 0.990 |
|  | *MTT3* | -3.604 | 89.4 | 12.027 | 0.992 |
|  | *MTT5* | -3.701 | 86.3 | 7.231 | 0.989 |

(*): correlation coefficient. Efficiency (E) is calculated from the slope value of the standard curve: E = 10^(-1/slope)-1^
