# Supplementary material for: Extreme metal adapted, knockout and knockdown strains reveal a coordinated gene expression among different Tetrahymena thermophila metallothionein isoforms
Source: PLoS One. 2017 Dec 5;12(12):e0189076. doi: 10.1371/journal.pone.0189076 (PMC5716537; doi:10.1371/journal.pone.0189076)
Supplement: S3 Table — (-): Data not obtained. Normalization of the gene expression was carried out using the β-actin as an endogenous control gene. We show the average value ± standard deviation of two or three independent experiments. (-1M) or (-6M): metal adapted strains after 1 or 6 months in growth medium without metal exposure. (DOCX) [file pone.0189076.s004.docx]

**S3 Table.** MT gene induction values obtained by qRT-PCR after different metal treatments

| ***MTT1* gene** | | | | | | | | | |
| --- | --- | --- | --- | --- | --- | --- | --- | --- | --- |
| **Strain** | **Cd 1h** | **Cd 24h** | **CdMTC** | **Cu 1h** | **Cu 24h** | **CuMTC** | **Pb 1h** | **Pb 24h** | **PbMTC** |
| **Control** | 156.31 ± 51.64 | 247.14 ± 51.19 | - | 3.29 ± 0.99 | 1.53 ± 0.1 | - | 16.11 ± 3.61 | 20.19 ± 2.23 | - |
| **Cd-adap** | 5.91 ± 0.85 | 23.56 ± 2.31 | 80.22 ± 16.5 | 13.21 ± 3.9 | 5.6 ± 0.15 | - | 2.18 ± 0.21 | 1.24 ± 0.14 | - |
| **Cu-adap** | 151.58 ± 22.69 | 341.74 ± 69.9 | - | 96.1 ± 17.97 | 39.1 ± 7.4 | 9.4 ± 1.65 | 6.33 ± 0.72 | 19.48 ± 6.13 | - |
| **Pb-adap** | 158.82 ± 3.75 | 231.95 ± 11.89 | - | 90.97 ± 19.7 | 40.03 ± 13.5 | - | 4.38 ± 0.56 | 4.43 ± 0.86 | 13.25 ± 1.15 |
| **Adap strains (-1M)** | 4,049.05 ± 590.9 | 6,895.7±1,024 | - | 32.13 ± 11.5 | 7.32 ± 0.28 | - | 5.54 ± 0.25 | 8.83 ± 2.08 | - |
| **Adap strains (-6M)** | 89.79 ± 36.3 | 448.42 ± 24.26 | - | 10.15 ± 2.32 | 17.69 ± 11.3 | - | 8.76 ± 1.14 | 45.98 ± 1.74 | - |
| **GFPMTT5** | 7.74 ± 0.73 | 36.32 ± 5.56 | - | 9.55 ± 1.94 | 2.8 ± 0.35 | - | 2.45 ± 0.31 | 2.16 ± 0.3 | - |
| **GFPMTT1** | 21.82 ± 1.51 | 44.51 ± 6.57 | - | 4.32 ± 1.21 | 2.63 ± 0.4 | - | 0.15 ± 0.01 | 11.74 ± 3.17 | - |
| **MTT5KD** | 135.06 ± 43.02 | 290.29 ± 86.59 | - | 10.62 ± 1.05 | 5.89 ± 1.39 | - | 22.43 ± 0.11 | 30.7 ± 5.23 | - |
| ***MTT3* gene** | | | | | | | | | |
| **Strain** | **Cd 1h** | **Cd 24h** | **CdMTC** | **Cu 1h** | **Cu 24h** | **CuMTC** | **Pb 1h** | **Pb 24h** | **PbMTC** |
| **Control** | 14.04 ± 4.5 | 21.65 ± 4.56 | - | 1.75 ± 0.2 | 1.43 ± 0.07 | - | 17.81 ± 3.38 | 4.33 ± 0.31 | - |
| **Cd-adap** | 21.5 ± 2.44 | 32.25 ± 3.32 | 32.69 ± 1.99 | 52.02 ± 1.06 | 137.2 ± 5.96 | - | 2.69 ± 0.27 | 1.03 ± 0.11 | - |
| **Cu-adap** | 61.73 ± 4.05 | 346.82 ± 77.2 | - | 86.44 ± 13.6 | 34.9 ± 2.56 | 3.45 ± 0.43 | 0.94 ± 0.11 | 9.54 ± 1.83 | - |
| **Pb-adap** | 98.46 ± 11.28 | 157.86 ± 6.85 | - | 130.5 ± 30.9 | 16.19 ± 2.37 | - | 3.62 ± 0.17 | 1.85 ± 0.1 | 2.85 ± 0.25 |
| **Adap strains (-1M)** | 204.06 ± 29.87 | 626.72 ± 130.5 | - | 64.4 ± 23.04 | 4.02 ± 0.21 | - | 10.19 ± 0.89 | 10.43 ± 1.51 | - |
| **Adap strains (-6M)** | 134.5 ± 12.55 | 170.32 ± 14.37 | - | 67.11 ± 16.8 | 30.19 ± 5.24 | - | 0.27 ± 0.03 | 6.37 ± 0.13 | - |
| **GFPMTT5** | 41.9 ± 2.17 | 99.45 ± 23.73 | - | 24.82 ± 0.63 | 13.38 ± 1.66 | - | 4.16 ± 0.17 | 6.19 ± 0.85 | - |
| **GFPMTT1** | 13.04 ± 2.99 | 31.77 ± 4.27 | - | 9.91 ± 2.64 | 4.17 ± 1.82 | - | 0.1 ± 0.07 | 0.71 ± 0.08 | - |
| **MTT1KO** | 24.93 ± 0.6 | 127.89 ± 2.94 | - | 2.65 ± 0.45 | 8.99 ± 0.71 | - | 13.2 ± 1.79 | 6.55 ± 2.74 | - |
| **MTT5KD** | 79.35 ± 14.22 | 200.05 ± 58.5 | - | 9.004 ± 1.01 | 5.44 ± 1.09 | - | 13.45 ± 0.99 | 26.15 ± 4.58 | - |
| **MTT1KO + MTT5KD** | 15.55 ± 1.12 | 36.65 ± 3.45 | - | 2.01 ± 0.07 | 0.56 ± 0.06 | - | 11.34 ± 0.13 | 5.75 ± 0.51 | - |

| ***MTT5* gene** | | | | | | | | | |
| --- | --- | --- | --- | --- | --- | --- | --- | --- | --- |
| **Strain** | **Cd 1h** | **Cd 24h** | **CdMTC** | **Cu 1h** | **Cu 24h** | **CuMTC** | **Pb 1h** | **Pb 24h** | **PbMTC** |
| **Control** | 863.01±240.85 | 2,056.3±297.5 | - | 49.4 ± 14.17 | 38.11 ± 3.28 | - | 192.2±48.35 | 4,212.7±773.5 | - |
| **Cd-adap** | 38.44 ± 16.41 | 80.13 ± 1.57 | 420.2 ± 81.4 | 45.09 ± 7.8 | 12.06 ± 1.3 | - | 37.2 ± 3.66 | 136.21 ± 9.53 | - |
| **Cu-adap** | 192.1 ± 33.09 | 930.46±189.87 | - | 113.2 ± 27.8 | 25.58 ± 4.96 | 47.5 ± 6.06 | 53.79 ± 1.06 | 271.47±68.71 | - |
| **Pb-adap** | 323.74 ± 10.99 | 336.59 ± 63.17 | - | 359.6± 171.1 | 238.2 ± 20.5 | - | 22.25 ± 2.87 | 60.64 ± 19.24 | 556.4±114.1 |
| **Adap strains (-1M)** | 231.65 ± 33.75 | 15,429.9±2,427.7 | - | 18.28 ± 5.16 | 13.23 ± 1.68 | - | 7.89 ± 0.09 | 16.97 ± 5.48 | - |
| **Adap strains (-6M)** | 162.45 ± 11.12 | 523.22 ± 131.96 | - | 11.99 ± 2.76 | 20.89 ± 5.54 | - | 23.29 ± 4.91 | 626.71 ± 23.97 | - |
| **GFPMTT5** | 50.39 ± 8.9 | 1,579.2±358.3 | - | 5.82 ± 0.33 | 5.11 ± 0.62 | - | 12.74 ± 1.46 | 23.06 ± 7.97 | - |
| **GFPMTT1** | 170,47 ± 5.87 | 537.1 ± 79.58 | - | 4.33 ± 1.38 | 53.48 ± 7.69 | - | 9.13 ± 0.91 | 1,171.2±297.6 | - |
| **MTT1KO** | 544.82 ± 110.4 | 703.08 ± 49.7 | - | 24.38 ± 3.81 | 23.72 ± 1.71 | - | 246.97 ±32.5 | 454.82±145.58 | - |
| **MTT5KD** | 617.68 ± 137.8 | 3,967.1±1,100.9 | - | 48.74 ± 4.6 | 117.2 ± 23.6 | - | 302.1±12.42 | 3,747.6 ±690.9 | - |
| **MTT1KO + MTT5KD** | 42.89 ± 4.82 | 76.57 ± 1.34 | - | 2.09 ± 0.09 | 2.45 ± 0.41 | - | 20.52 ± 0.5 | 108.4 ± 16.93 | - |
| ***MTT2/4* genes** | | | | | | | | | |
| **Strain** | **Cd 1h** | **Cd 24h** | **CdMTC** | **Cu 1h** | **Cu 24h** | **CuMTC** | **Pb 1h** | **Pb 24h** | **PbMTC** |
| **Control** | 37.53 ± 10.46 | 20.57 ± 3.82 | - | 46.9 ± 11.12 | 25.66 ± 3.24 | - | 9.46 ± 2.2 | 2.79 ± 0.3 | - |
| **Cd-adap** | 1.86 ± 0.39 | 2.67 ±0.14 | 2.29 ± 0.62 | 307.2 ± 99.6 | 99.61 ± 2.35 | - | 2.83 ± 0.57 | 0.69 ± 0.11 | - |
| **Cu-adap** | 5.01 ± 0.52 | 7.23 ± 1.55 | - | 41.4 ± 7.2 | 8.73 ± 1.73 | 4.77 ± 0.6 | 1.73 ± 0.27 | 0.4 ± 0.08 | - |
| **Pb-adap** | 6.35 ± 0.26 | 20.26 ± 0.97 | - | 326.3 ± 71.6 | 89.6 ± 9.35 | - | 0.36 ± 0.02 | 0.5 ± 0.1 | 0.85 ± 0.09 |
| **Adap strains (-1M)** | 34.68 ± 6.73 | 15.57 ± 2.33 | - | 93.7 ± 19.31 | 5.68 ± 0.91 | - | 5.41 ± 0.36 | 0.95 ± 0.24 | - |
| **Adap strains (-6M)** | 60.87 ± 3.94 | 89.55 ± 5.73 | - | 40.52 ± 9.26 | 43.7 ± 11.33 | - | 2.95 ± 0.25 | 2.25 ± 0.04 | - |
| **GFPMTT5** | 2.32 ± 0.2 | 1.68 ± 0.24 | - | 103.2 ± 1.71 | 19.45 ± 2.35 | - | 3.27 ± 0.28 | 0.38 ± 0.05 | - |
| **GFPMTT1** | 4.62 ± 0.9 | 1.28 ± 0.26 | - | 44.68 ± 13.3 | 6.86 ± 0.62 | - | 0.06 ± 0.01 | 0.28 ± 0.02 | - |
| **MTT1KO** | 18.42 ± 0.51 | 12.59 ± 0.32 | - | 37.04 ± 5.68 | 40.94 ± 5.48 | - | 7.71 ± 1.53 | 0.29 ± 0.1 | - |
| **MTT5KD** | 12.19 ± 2.12 | 14.58 ± 4.05 | - | 48.97 ± 7.73 | 28.28 ± 6.01 | - | 5.43 ± 0.68 | 1.08 ± 0.19 | - |
| **MTT1KO + MTT5KD** | 11.53 ± 0.85 | 3.93 ± 0.18 | - | 9.38 ± 0.5 | 5.29 ± 0.86 | - | 2.96 ± 0.09 | 1.32 ± 0.13 | - |

(-): Data not obtained. Normalization of the gene expression was carried out using the β-actin as an endogenous control gene. We show the average value ± standard deviation of two or three independent experiments. (-1M) or (-6M): metal adapted strains after 1 or 6 months in growth medium without metal exposure.
