# Supplementary material for: Extreme metal adapted, knockout and knockdown strains reveal a coordinated gene expression among different Tetrahymena thermophila metallothionein isoforms
Source: PLoS One. 2017 Dec 5;12(12):e0189076. doi: 10.1371/journal.pone.0189076 (PMC5716537; doi:10.1371/journal.pone.0189076)
Supplement: S4 Table — β-actin gene was used as an endogenous control and it was considered a reference for neutralizing the variability of the qPCR technique. (*) Ct values that are considerably lower than those obtained in the control SB1969 strain. (-): not applicable, because the MTT1KO and the MTT1KO + MTT5KD strains have lost all the copies of the MTT1 gene. (-1M) or (-6M): these parameters were calculated after maintaining metal adapted strains 1 or 6 months in growth medium without metal exposure. (DOCX) [file pone.0189076.s005.docx]

**S4 Table**. Comparison of the C_t_ values obtained in control situations (no metal exposure) from the different *T. thermophila* strains analyzed in the comparative MT gene expression study

| **Gene** | **Control SB1969** | **Cd-adap** | **Cu-adap** | **Pb-adap** | **GFPMTT1** | **GFPMTT5** | **MTT1KO** | **MTT5KD** | **MTT1KO + MTT5KD** |
| --- | --- | --- | --- | --- | --- | --- | --- | --- | --- |
| ***β-actin*** | 14.95 | 15.6 | 14.80 | 15.10 | 14.90 | 15.00 | 13.1 | 15.6 | 13.8 |
| ***MTT1*** | 16.42 | 11.61* | 17.40 | 17.50 | 15.41 | 14.23* | - | 17.5 | - |
| ***MTT3*** | 17.10 | 16.50 | 19.30 | 20.40 | 17.20 | 18.70 | 17.3 | 22.6 | 18.3 |
| ***MTT5*** | 21.59 | 17.70* | 20.10* | 18.70* | 20.52 | 15.98* | 18.5* | 28.6 | 15.7* |
| ***MTT2/4*** | 19.47 | 18.50 | 15.34* | 19.20 | 16.67* | 16.79* | 16.5* | 18.7 | 16.1* |
| **Gene** | **Cd-adap**  **(-1M)** | **Cu- adap**  **(- 1M)** | **Pb- adap**  **(-1M)** | **Cd- adap**  **(-6M)** | **Cu- adap**  **(-6M)** | **Pb- adap (-6M)** |  | | |
| ***β-actin*** | 14.80 | 14.60 | 16.10 | 14.70 | 16.30 | 14.90 |  |  |  |
| ***MTT1*** | 17.20 | 17.60 | 15.60 | 16.50 | 16.40 | 15.70 |  |  |  |
| ***MTT3*** | 20.30 | 20.20 | 20.2 | 19.50 | 19.90 | 19.20 |  |  |  |
| ***MTT5*** | 19.00* | 20.90 | 14.20* | 19.20* | 18.50* | 17.20* |  |  |  |
| ***MTT2/4*** | 17.60* | 17.50* | 19.30 | 19.90 | 18.30* | 19.30 |  |  |  |

*β-actin* gene was used as an endogenous control and it was considered a reference for neutralizing the variability of the qPCR technique. ^(^*^)^ C_t_ values that are considerably lower than those obtained in the control SB1969 strain. (-): not applicable, because the MTT1KO and the MTT1KO + MTT5KD strains have lost all the copies of the *MTT1* gene. (-1M) or (-6M): these parameters were calculated after maintaining metal adapted strains 1 or 6 months in growth medium without metal exposure.
