# Supplementary material for: Extreme metal adapted, knockout and knockdown strains reveal a coordinated gene expression among different Tetrahymena thermophila metallothionein isoforms
Source: PLoS One. 2017 Dec 5;12(12):e0189076. doi: 10.1371/journal.pone.0189076 (PMC5716537; doi:10.1371/journal.pone.0189076)
Supplement: S5 Table — Differences among basal expression levels for the different MT genes in each T. thermophila strain were calculated using the following formula: 2(Ct1-Ct2), being Ct1 and Ct2 the Ct values under a control situation (no metal exposure) between two MT genes in the same strain. We compared in each strain all MT gene basal expression levels by twos, distinguishing them by two colours: red and green. For each comparison, results are indicated in red or green depending on the MT gene that has a higher basal expression level in the same strain. Comparison values higher than 4 (Ct value differences higher than 2 cycles) are shaded in grey. (-): not applicable. (-1M) or (-6M): these parameters were calculated after maintaining metal adapted strains 1 or 6 months in growth medium without metal exposure. (DOCX) [file pone.0189076.s006.docx]

**S5 Table.** Comparison of the basal expression levels among different MT gene isoforms in each *T. thermophila* strain

| **SB1969** | ***MTT1*** | ***MTT3*** | ***MTT5*** | ***MTT2/4*** | **Cd-adap** | ***MTT1*** | ***MTT3*** | ***MTT5*** | ***MTT2/4*** |
| --- | --- | --- | --- | --- | --- | --- | --- | --- | --- |
| ***MTT1*** | **1** |  |  |  | ***MTT1*** | **1** |  |  |  |
| ***MTT3*** | **1.60x** | **1** |  |  | ***MTT3*** | **29.65x** | **1** |  |  |
| ***MTT5*** | **36x** | **22.47x** | **1** |  | ***MTT5*** | **68.12x** | **2.3x** | **1** |  |
| ***MTT2/4*** | **8.28x** | **5.17x** | **4.35x** | **1** | ***MTT2/4*** | **118.6x** | **4x** | **1.74x** | **1** |
| **Cd-adap**  **(-1M)** | ***MTT1*** | ***MTT3*** | ***MTT5*** | ***MTT2/4*** | **Cd-adap**  **(- 6M)** | ***MTT1*** | ***MTT3*** | ***MTT5*** | ***MTT2/4*** |
| ***MTT1*** | **1** |  |  |  | ***MTT1*** | **1** |  |  |  |
| ***MTT3*** | **8.57x** | **1** |  |  | ***MTT3*** | **8x** | **1** |  |  |
| ***MTT5*** | **3.48x** | **2.46x** | **1** |  | ***MTT5*** | **6.5x** | **1.23x** | **1** |  |
| ***MTT2/4*** | **1.32x** | **6.5x** | **2.64x** | **1** | ***MTT2/4*** | **10.56x** | **1.32x** | **1.62x** | **1** |
| **Cu-adap** | ***MTT1*** | ***MTT3*** | ***MTT5*** | ***MTT2/4*** | **Cu-adap**  **(- 1M)** | ***MTT1*** | ***MTT3*** | ***MTT5*** | ***MTT2/4*** |
| ***MTT1*** | **1** |  |  |  | ***MTT1*** | **1** |  |  |  |
| ***MTT3*** | **3.73x** | **1** |  |  | ***MTT3*** | **6.06x** | **1** |  |  |
| ***MTT5*** | **6.5x** | **1.74x** | **1** |  | ***MTT5*** | **9.85x** | **1.62x** | **1** |  |
| ***MTT2/4*** | **4.17x** | **15.56x** | **27.1x** | **1** | ***MTT2/4*** | **1.07x** | **6.5x** | **10.56x** | **1** |
| **Cu-adap**  **(-6M)** | ***MTT1*** | ***MTT3*** | ***MTT5*** | ***MTT2/4*** | **Pb-adap** | ***MTT1*** | ***MTT3*** | ***MTT5*** | ***MTT2/4*** |
| ***MTT1*** | **1** |  |  |  | ***MTT1*** | **1** |  |  |  |
| ***MTT3*** | **11.3x** | **1** |  |  | ***MTT3*** | **7.46x** | **1** |  |  |
| ***MTT5*** | **4.29x** | **2.64x** | **1** |  | ***MTT5*** | **2.3x** | **3.25x** | **1** |  |
| ***MTT2/4*** | **3.73x** | **3.03x** | **1.15x** | **1** | ***MTT2/4*** | **3.25x** | **2.3x** | **1.41x** | **1** |

| **Pb-adap**  **(-1M)** | ***MTT1*** | ***MTT3*** | ***MTT5*** | ***MTT2/4*** | **Pb-adap**  **(- 6M)** | ***MTT1*** | ***MTT3*** | ***MTT5*** | ***MTT2/4*** |
| --- | --- | --- | --- | --- | --- | --- | --- | --- | --- |
| ***MTT1*** | **1** |  |  |  | ***MTT1*** | **1** |  |  |  |
| ***MTT3*** | **24.25x** | **1** |  |  | ***MTT3*** | **11.3x** | **1** |  |  |
| ***MTT5*** | **2.64x** | **64x** | **1** |  | ***MTT5*** | **2.83x** | **4x** | **1** |  |
| ***MTT2/4*** | **12.99x** | **1.87x** | **34.3x** | **1** | ***MTT2/4*** | **12.13x** | **1.07x** | **4.29x** | **1** |
| **GFPMTT5** | ***MTT1*** | ***MTT3*** | ***MTT5*** | ***MTT2/4*** | **GFPMTT1** | ***MTT1*** | ***MTT3*** | ***MTT5*** | ***MTT2/4*** |
| ***MTT1*** | **1** |  |  |  | ***MTT1*** | **1** |  |  |  |
| ***MTT3*** | **22.16x** | **1** |  |  | ***MTT3*** | **3.46x** | **1** |  |  |
| ***MTT5*** | **3.36x** | **6.59x** | **1** |  | ***MTT5*** | **34.54x** | **9.99x** | **1** |  |
| ***MTT2/4*** | **5.9x** | **3.76x** | **1.75x** | **1** | ***MTT2/4*** | **2.39x** | **1.44x** | **3.85x** | **1** |
| **MTT1KO** | ***MTT1*** | ***MTT3*** | ***MTT5*** | ***MTT2/4*** | **MTT5KD** | ***MTT1*** | ***MTT3*** | ***MTT5*** | ***MTT2/4*** |
| ***MTT1*** | **-** |  |  |  | ***MTT1*** | **1** |  |  |  |
| ***MTT3*** | **-** | **1** |  |  | ***MTT3*** | **34.3x** | **1** |  |  |
| ***MTT5*** | **-** | **2.3x** | **1** |  | ***MTT5*** | **2,194.9x** | **64x** | **1** |  |
| ***MTT2/4*** | **-** | **1.74x** | **4x** | **1** | ***MTT2/4*** | **2.3x** | **14.93x** | **955.4x** | **1** |
| **MTT1KO+**  **MTT5KD** | ***MTT1*** | ***MTT3*** | ***MTT5*** | ***MTT2/4*** |  | | | | |
| ***MTT1*** | **-** |  |  |  |  |  |  |  |  |
| ***MTT3*** | **-** | **1** |  |  |  |  |  |  |  |
| ***MTT5*** | **-** | **6.06x** | **1** |  |  |  |  |  |  |
| ***MTT2/4*** | **-** | **4.59x** | **1.32x** | **1** |  |  |  |  |  |

Differences among basal expression levels for the different MT genes in each *T. thermophila* strain were calculated using the following formula: 2^(Ct1-Ct2)^, being C_t1_ and C_t2_ the C_t_ values under a control situation (no metal exposure) between two MT genes in the same strain. We compared in each strain all MT gene basal expression levels by twos, distinguishing them by two colours: red and green. For each comparison, results are indicated in red or green depending on the MT gene that has a higher basal expression level in the same strain. Comparison values higher than 4 (C_t_ value differences higher than 2 cycles) are shaded in grey. (-): not applicable. (-1M) or (- 6M): these parameters were calculated after maintaining metal adapted strains 1 or 6 months in growth medium without metal exposure.
