# Supplementary material for: Extreme metal adapted, knockout and knockdown strains reveal a coordinated gene expression among different Tetrahymena thermophila metallothionein isoforms
Source: PLoS One. 2017 Dec 5;12(12):e0189076. doi: 10.1371/journal.pone.0189076 (PMC5716537; doi:10.1371/journal.pone.0189076)
Supplement: S6 Table — Differences among basal expression levels for each MT gene among different T. thermophila strains were calculated using the following formula: 2(Ct1-Ct2), being Ct1 and Ct2 the Ct values under a control situation (no metal exposure) for the same MT gene into two different strains. We compared all the T. thermophila analyzed strains by twos, distinguishing them by two colours: red and green. For each comparison, results are indicated in red or green depending on the strain that has a higher basal expression level for the same MT gene. Comparison values which are higher than 4 (Ct value differences higher than 2 cycles) are shaded in grey. (-): not applicable. (-1M) or (-6M): these parameters were calculated after maintaining metal adapted strains 1 or 6 months in growth medium without metal exposure. (DOCX) [file pone.0189076.s007.docx]

**S6 Table.** Comparison of the basal expression levels of each MT gene among different *T. thermophila* strains

| ***MTT1* gene** | **SB1969** | **Cd-adap** | **Cd-adap**  **(-1M)** | **Cd-adap**  **(-6M)** | **Cu-adap** | **Cu-adap**  **(-1M)** | **Cu-adap**  **(-6M)** | **Pb-adap** | **Pb-adap**  **(-1M)** | **Pb-adap**  **(-6M)** | **GFP**  **MTT5** | **GFP**  **MTT1** | **MTT1KO** | **MTT5**  **KD** | **MTT1KO + MTT5KD** |
| --- | --- | --- | --- | --- | --- | --- | --- | --- | --- | --- | --- | --- | --- | --- | --- |
| **SB1969** | **1** |  |  |  |  |  |  |  |  |  |  |  |  |  |  |
| **Cd-adap** | **28.05x** | **1** |  |  |  |  |  |  |  |  |  |  |  |  |  |
| **Cd-adap**  **(-1M)** | **1.72x** | **48.2x** | **1** |  |  |  |  |  |  |  |  |  |  |  |  |
| **Cd-adap**  **(-6M)** | **1.06x** | **29.7x** | **1.62x** | **1** |  |  |  |  |  |  |  |  |  |  |  |
| **Cu-adap** | **1.97x** | **55.3x** | **1.15x** | **1.87x** | **1** |  |  |  |  |  |  |  |  |  |  |
| **Cu-adap**  **(-1M)** | **2.26x** | **63.6x** | **1.32x** | **2.14x** | **1.15x** | **1** |  |  |  |  |  |  |  |  |  |
| **Cu-adap**  **(-6M)** | **1.01x** | **27.7x** | **1.74x** | **1.07x** | **2x** | **2.29x** | **1** |  |  |  |  |  |  |  |  |
| **Pb-adap** | **2.11x** | **59.3x** | **1.23x** | **2x** | **1.07x** | **1.07x** | **2.14x** | **1** |  |  |  |  |  |  |  |
| **Pb-adap**  **(-1M)** | **1.77x** | **15.9x** | **3.03x** | **1.87x** | **3.48x** | **4x** | **1.74x** | **3.73x** | **1** |  |  |  |  |  |  |
| **Pb-adap**  **(-6M)** | **1.65x** | **17.0x** | **2.82x** | **1.74x** | **3.25x** | **3.73x** | **1.62x** | **3.48x** | **1.07** | **1** |  |  |  |  |  |
| **GFPMTT5** | **4.56x** | **6.14x** | **7.84x** | **4.82x** | **9.00x** | **10.34x** | **4.50x** | **9.65x** | **2.58x** | **2.78x** | **1** |  |  |  |  |
| **GFPMTT1** | **2.01x** | **13.9x** | **3.46x** | **2.13x** | **3.97x** | **4.56x** | **1.99x** | **4.26x** | **1.14x** | **1.22x** | **2.27x** | **1** |  |  |  |
| **MTT1KO** | **-** | - | **-** | **-** | **-** | **-** | **-** | **-** | **-** | **-** | **-** | **-** | **1** |  |  |
| **MTT5KD** | **2.11x** | **59.3x** | **1.23x** | **2x** | **1.07x** | **1.07x** | **2.14x** | **1x** | **3.73x** | **3.48x** | **9.65x** | **4.26x** | - | **1** |  |
| **MTT1KO +**  **MTT5KD** | - | - | - | **-** | **-** | - | - | - | - | - | - | - | - | - | **1** |

| ***MTT3***  **gene** | **SB1969** | **Cd-adap** | **Cd-adap**  **(-1M)** | **Cd-adap**  **(-6M)** | **Cu-adap** | **Cu-adap**  **(-1M)** | **Cu-adap**  **(-6M)** | **Pb-adap** | **Pb-adap**  **(-1M)** | **Pb-adap**  **(-6M)** | **GFP**  **MTT5** | **GFP**  **MTT1** | **MTT1KO** | **MTT5**  **KD** | **MTT1KO + MTT5KD** |
| --- | --- | --- | --- | --- | --- | --- | --- | --- | --- | --- | --- | --- | --- | --- | --- |
| **SB1969** | **1** |  |  |  |  |  |  |  |  |  |  |  |  |  |  |
| **Cd-adap** | **1.52x** | **1** |  |  |  |  |  |  |  |  |  |  |  |  |  |
| **Cd-adap**  **(-1M)** | **9.19x** | **13.9x** | **1** |  |  |  |  |  |  |  |  |  |  |  |  |
| **Cd-adap**  **(-6M)** | **5.28x** | **8x** | **1.74x** | **1** |  |  |  |  |  |  |  |  |  |  |  |
| **Cu-adap** | **4.59x** | **6.96x** | **2x** | **1.15x** | **1** |  |  |  |  |  |  |  |  |  |  |
| **Cu-adap**  **(-1M)** | **8.57x** | **13x** | **1.07x** | **1.62x** | **1.87x** | **1** |  |  |  |  |  |  |  |  |  |
| **Cu-adap**  **(-6M)** | **6.96x** | **10.6x** | **1.32x** | **1.32x** | **1.52x** | **1.23x** | **1** |  |  |  |  |  |  |  |  |
| **Pb-adap** | **9.85x** | **14.9x** | **1.07x** | **1.87x** | **2.14x** | **1.15x** | **1.41x** | **1** |  |  |  |  |  |  |  |
| **Pb-adap**  **(-1M)** | **8.57x** | **13x** | **1.07x** | **1.62x** | **1.87x** | **1x** | **1.23x** | **1.15x** | **1** |  |  |  |  |  |  |
| **Pb-adap**  **(-6M)** | **4.28x** | **6.49x** | **1.87x** | **1.23x** | **1.07x** | **2x** | **1.62x** | **2.3x** | **2x** | **1** |  |  |  |  |  |
| **GFPMTT5** | **3.03x** | **4.59x** | **3.03x** | **1.74x** | **1.52x** | **2.83x** | **2.3x** | **3.25x** | **2.83x** | **1.41x** | **1** |  |  |  |  |
| **GFPMTT1** | **1.07x** | **1.62x** | **8.58x** | **4.92x** | **4.29x** | **8x** | **6.5x** | **9.19x** | **8x** | **4x** | **2.82x** | **1** |  |  |  |
| **MTT1KO** | **1.15x** | **1.74x** | **8x** | **4.59x** | **4x** | **7.46x** | **6.06x** | **8.57x** | **7.46x** | **3.73x** | **2.63x** | **1.07x** | **1** |  |  |
| **MTT5KD** | **42.2x** | **64x** | **4.59x** | **8x** | **9.19x** | **4.92x** | **6.06x** | **4.29x** | **4.93x** | **9.85x** | **13.9x** | **39.4x** | **36.7x** | **1** |  |
| **MTT1KO +**  **MTT5KD** | **2.3x** | **3.48x** | **4x** | **2.3x** | **2x** | **3.73x** | **3.03x** | **4.29x** | **3.73x** | **1.87x** | **1.32x** | **2.14x** | **2x** | **18.4x** | **1** |

| ***MTT5***  **gene** | **SB1969** | **Cd-adap** | **Cd-adap**  **(-1M)** | **Cd-adap**  **(-6M)** | **Cu-adap** | **Cu-adap**  **(-1M)** | **Cu-adap**  **(-6M)** | **Pb-adap** | **Pb-adap**  **(-1M)** | **Pb-adap**  **(-6M)** | **GFP**  **MTT5** | **GFP**  **MTT1** | **MTT1**  **KO** | **MTT5**  **KD** | **MTT1KO + MTT5KD** |
| --- | --- | --- | --- | --- | --- | --- | --- | --- | --- | --- | --- | --- | --- | --- | --- |
| **SB1969** | **1** |  |  |  |  |  |  |  |  |  |  |  |  |  |  |
| **Cd-adap** | **14.83** | **1** |  |  |  |  |  |  |  |  |  |  |  |  |  |
| **Cd-adap**  **(-1M)** | **6.02x** | **2.46x** | **1** |  |  |  |  |  |  |  |  |  |  |  |  |
| **Cd-adap**  **(-6M)** | **5.24x** | **2.83x** | **1.15 x** | **1** |  |  |  |  |  |  |  |  |  |  |  |
| **Cu-adap** | **2.81x** | **5.28x** | **2.14x** | **1.87x** | **1** |  |  |  |  |  |  |  |  |  |  |
| **Cu-adap**  **(-1M)** | **1.61x** | **9.19x** | **3.73x** | **3.25x** | **1.74x** | **1** |  |  |  |  |  |  |  |  |  |
| **Cu-adap**  **(-6M)** | **8.51x** | **1.74x** | **1.41x** | **1.62x** | **3.03x** | **5.28x** | **1** |  |  |  |  |  |  |  |  |
| **Pb-adap** | **7.41** | **2x** | **1.23x** | **1.14x** | **2.64x** | **4.59x** | **1.15x** | **1** |  |  |  |  |  |  |  |
| **Pb-adap**  **(-1M)** | **167.7x** | **11.3x** | **27.86x** | **32x** | **59.7x** | **103.97x** | **19.7x** | **22.6x** | **1** |  |  |  |  |  |  |
| **Pb-adap**  **(-6M)** | **20.97x** | **1.41x** | **3.48x** | **4x** | **7.46x** | **12.99x** | **2.46x** | **2.83x** | **8x** | **1** |  |  |  |  |  |
| **GFPMTT5** | **48.84x** | **3.29x** | **8x** | **9.32x** | **17.39x** | **30.27x** | **5.74x** | **6.59x** | **3.43x** | **2.32x** | **1** |  |  |  |  |
| **GFPMTT1** | **2.09x** | **7.06x** | **2.8x** | **2.5x** | **1.34x** | **1.34x** | **4.06x** | **3.53x** | **79.89x** | **9.99x** | **23.26x** | **1** |  |  |  |
| **MTT1KO** | **8.51x** | **1.74x** | **1.41x** | **1.62x** | **3.03x** | **5.28x** | **1x** | **1.15x** | **19.7x** | **2.46x** | **5.74x** | **4.06x** | **1** |  |  |
| **MTT5KD** | **120.3x** | **1,783x** | **724x** | **630.3x** | **337.8x** | **194x** | **1,024x** | **891.4x** | **20,171x** | **2,521.4x** | **5,873x** | **252.5x** | **1,024x** | **1** |  |
| **MTT1KO +**  **MTT5KD** | **59.3x** | **4x** | **9.85x** | **11.31x** | **21.1x** | **36.76x** | **6.96x** | **8x** | **2.83x** | **2.83x** | **1.21x** | **28.25x** | **6.96x** | **7,131x** | **1** |

| ***MTT2/4***  **genes** | **SB1969** | **Cd-adap** | **Cd-adap**  **(-1M)** | **Cd-adap**  **(-6M)** | **Cu-adap** | **Cu-adap**  **(-1M)** | **Cu-adap**  **(-6M)** | **Pb-adap** | **Pb-adap**  **(-1M)** | **Pb-adap**  **(-6M)** | **GFP**  **MTT5** | **GFP**  **MTT1** | **MTT1KO** | **MTT5**  **KD** | **MTT1KO + MTT5KD** |
| --- | --- | --- | --- | --- | --- | --- | --- | --- | --- | --- | --- | --- | --- | --- | --- |
| **SB1969** | **1** |  |  |  |  |  |  |  |  |  |  |  |  |  |  |
| **Cd-adap** | **1.94x** | **1** |  |  |  |  |  |  |  |  |  |  |  |  |  |
| **Cd-adap**  **(-1M)** | **3.66x** | **1.87x** | **1** |  |  |  |  |  |  |  |  |  |  |  |  |
| **Cd-adap**  **(-6M)** | **1.35x** | **2.64x** | **4.92x** | **1** |  |  |  |  |  |  |  |  |  |  |  |
| **Cu-adap** | **17.51x** | **8.94x** | **4.79x** | **23.6x** | **1** |  |  |  |  |  |  |  |  |  |  |
| **Cu-adap**  **(-1M)** | **3.92** | **2x** | **1.07x** | **5.28x** | **4.47x** | **1** |  |  |  |  |  |  |  |  |  |
| **Cu-adap**  **(-6M)** | **2.25x** | **1.15x** | **1.62x** | **3.03x** | **7.78x** | **1.74x** | **1** |  |  |  |  |  |  |  |  |
| **Pb-adap** | **1.62x** | **1.62x** | **3.03x** | **1.62x** | **14.5x** | **3.25x** | **1.87x** | **1** |  |  |  |  |  |  |  |
| **Pb-adap**  **(-1M)** | **1.13x** | **1.74x** | **3.25x** | **1.52x** | **15.6x** | **3.48x** | **2x** | **1.07x** | **1** |  |  |  |  |  |  |
| **Pb-adap**  **(-6M)** | **1.13x** | **1.74x** | **3.25x** | **1.52x** | **15.6x** | **3.48x** | **2x** | **1.07x** | **1x** | **1** |  |  |  |  |  |
| **GFPMTT5** | **6.41x** | **3.27x** | **1.75x** | **8.63x** | **2.73x** | **1.65x** | **2.85x** | **5.31x** | **5.7x** | **5.7x** | **1** |  |  |  |  |
| **GFPMTT1** | **6.96x** | **3.56x** | **1.91x** | **9.38x** | **2.51x** | **1.78x** | **3.1x** | **5.78x** | **6.19x** | **6.19x** | **1.09x** | **1** |  |  |  |
| **MTT1KO** | **7.84x** | **4x** | **2.14x** | **10.6x** | **2.23x** | **2x** | **3.48x** | **6.5x** | **6.95x** | **6.95x** | **1.22x** | **1.13x** | **1** |  |  |
| **MTT5KD** | **1.96x** | **1.15x** | **1.87x** | **2.64x** | **8.94x** | **2x** | **1.15x** | **1.62x** | **1.74x** | **1.74x** | **3.27x** | **3.56x** | **4x** | **1** |  |
| **MTT1KO+**  **MTT5KD** | **10.34x** | **5.28x** | **2.83x** | **13.93x** | **1.69x** | **2.64x** | **4.59x** | **8.57x** | **9.19x** | **9.19x** | **1.61x** | **1.48x** | **1.32x** | **5.28x** | **1** |

Differences among basal expression levels for each MT gene among different *T. thermophila* strains were calculated using the following formula: 2^(Ct1-Ct2)^, being C_t1_ and C_t2_ the C_t_ values under a control situation (no metal exposure) for the same MT gene into two different strains. We compared all the *T. thermophila* analyzed strains by twos, distinguishing them by two colours: red and green. For each comparison, results are indicated in red or green depending on the strain that has a higher basal expression level for the same MT gene. Comparison values which are higher than 4 (C_t_ value differences higher than 2 cycles) are shaded in grey. (-): not applicable. (-1M) or (- 6M): these parameters were calculated after maintaining metal adapted strains 1 or 6 months in growth medium without metal exposure.
